# Remarkable Wear Resistance in a Complex Concentrated Alloy with Nanohierarchical Architecture and Composition Undulation

**DOI:** 10.34133/research.0160

**Published:** 2023-06-05

**Authors:** Yushan Geng, Wenyuan Chen, Hui Tan, Jun Cheng, Shengyu Zhu, Jun Yang, Weimin Liu

**Affiliations:** ^1^ State Key Laboratory of Solid Lubrication, Lanzhou Institute of Chemical Physics, Chinese Academy of Sciences, Lanzhou 730000, PR China.; ^2^ Center of Materials Science and Optoelectronics Engineering, University of Chinese Academy of Sciences, Beijing 100049, PR China.; ^3^ Shandong Laboratory of Yantai Advanced Materials and Green Manufacturing, Yantai 264000, PR China.

## Abstract

Sustained wear damages on the sliding surfaces of alloys are generally the culprit responsible for the failure of various mechanical systems. Inspired by high-entropy effects, here we deliberately deploy nanohierarchical architecture with composition undulation in a Ni_50_(AlNbTiV)_50_ complex concentrated alloy, which yields ultralow wear rate within the order of 10^−7^ to 10^−6^ mm^3^/Nm between room temperature and 800 °C. Such remarkable wear resistance heretofore represents one of the highest wear resistance reported for the bulk alloys or composites, and originates from the multi-type adaptive friction interface protection governed by intrinsically nano-coupled grains and nanoprecipitates. This cooperative heterostructure releases gradient frictional stress in stages upon wear at room temperature through the coexistence of multiple deformation pathways while activating a dense nanocrystalline glaze layer upon wear at 800 °C to minimize adhesive and oxidative wear. Our work uncovers a practical avenue for tailoring wear properties with multicomponent heterostructures over a wide temperature range.

## Introduction

The wear-related interface failure severely limits the energy efficiency, service lifetime, and technological reliability of various structural and functional components [1,2]. Under thermomechanical environment interplay, sliding-induced deformation and unfavorable tribo-reaction always drive the metallic mating surface to suffer sustained damages ranging from surface roughening to localized fracture, rendering the tribo-systems almost unusable [3–5]. Pursuing ultra-wear-resistant alloys, with the wear rates generally below the order of 10^−5^ mm^3^/Nm over a wide temperature range, has long been a challenge for most advanced engineering applications [6].

Upon wear at low-to-moderate temperatures (<600 °C), where abrasive wear is the primary characteristic, the ability of the friction interface to withstand the elastic–plastic deformation determines the wear resistance of the material [2,7]. According to the classical Archard law [8], the wear resistance of the materials can be enhanced if their initial bearing capacities (hardness/yield strength) are improved, which suppresses crack initiation and propagation on the worn surface. Over the past decades, numerous investigations aimed at reducing wear have been carried out in a variety of nanostructured alloys with high hardness due to the Hall–Petch relation, but the results show that there is no absolute correlation between wear resistance and initial bearing capacities [1,2,9,10]. Nanograins possess very limited strain hardening ability so that plastic incompatibility and strain localization occur during surface folding and cracking, which result in wear particles, invariably evoking a dramatic micro-cutting and three-body abrasion [1,11]. Experimental results in recent years give an illuminating picture about the wear resistance of the alloys that can be elevated when introducing heterogeneous structural evolutions to accommodate strain gradients along the friction interface, e.g., by introducing nanocomposite/amorphous tribo-layers [2,12], precipitation-reinforced interfaces [3,13], gradient nanograined subsurfaces [1,4], friction-induced nano-twins [14,15], crystallographic textures [16], and self-organized lubricating tribo-layers [10,17]. In particular, recent studies on the wear response of complex concentrated alloys (CCAs) have advanced the understanding of wear-driven friction interface protection. For instance, in V_10_Cr_10_Fe_45_Co_30_Ni_5_ CCA [15] and TiZrHfTa_0.5_ CCA [18], as the applied load increases during sliding at room temperature (RT), including dislocation, mechanical nano-twins, heterostructures, and secondary phases are progressively activated for continued strengthening of the friction interface. Tribo-oxidation and contact shear can assist in the formation of reactive crystal-glass tribo-layers upon wear at RT and 300 °C for (TiNbZr)_75_Ag_25_ CCA [2] and TaMoNb CCA [7], respectively, so that the localized brittle fractures on the sliding surface are reversed to homogeneous deformation. Thus, if cooperative heterostructures capable of releasing frictional stress in stages are configured in the alloy, it is expected to suppress the sliding-induced deformation. In contrast, upon wear at high temperatures (≥600 °C), the wear behavior of the material becomes quite complex, stemming from the prevalence of surface adhesion and tribo-oxidation at the friction interface [19,20]. High-temperature sliding tends to soften the metallic surface that endows the near-surface material susceptible to chemical instability caused by counterbody materials or environmental oxygen, triggering severe delamination wear and composition segregation at the friction interface that exacerbates wear [21,22]. Materials become more dependent on tribochemical protection upon high-temperature wear due to deterioration in stiffness [17,23]. Tribo-oxidation may promote the formation of a protective oxide layer (glaze layer) consisting of various nanocrystalline (NC) oxides on the worn surface, which assists in enhancing the friction interface while providing solid lubrication [5,24,25]. If the worn surface is replaced by a glaze layer as tribo-oxidation accumulates, a gentle wear scenario can be achieved. Nevertheless, whether such glaze layer can be produced on the worn surface in time depends substantially on the sensitivity of the intrinsic structure in alloy matrix to tribochemical reactions. As for CCA, its wear resistance and thermal stability during high-temperature wear can be ameliorated by heterogeneous structural evolution. For example, coherent B2 nanoprecipitates and nano-layered pearlitic structure allow accelerated formation of protective glaze layers on worn TiMoNb CCA [5] and Fe_44.5_Co_25.4_Ni_24.4_Ti_5.7_ CCA [26] at 600 °C, respectively, while the Zener’s pinning effect associated with the (hetero-)interfaces can effectively suppress grain coarsening caused by sliding and heating.

Termed CCA or most famously as a subclass of these alloys known as high/medium-entropy alloys (H/MEAs), some of these alloys possess a variety of anomalous deformation mechanisms associated with multicomponent heterostructures, including saliently rugged local atomic-level pressures [27], chemical short-range order [28], spatially variable stacking faults (SFs) or twinning [29,30], severe lattice distortion [31], and extraordinary dislocation reaction [32]. This offers the possibility for the alloy to progressively release gradient frictional stress through the competition and coexistence of multiple deformation pathways during sliding at low-to-moderate temperatures. Meanwhile, the broad compositional space of the CCA system allows us to integrate various oxidation-prone alloying elements into the alloy matrix for the formation of protective glaze layers, minimizing the adhesive and oxidative wear between tribo-pairs during sliding at elevated temperatures. Inspired by the high-entropy effects, we replace minor alloying elements in nickel-based alloys with multicomponent alloying elements, which not only deliberately deploy nanohierarchical architecture with composition undulation in the system but also enhance the ability of the system to generate glaze layers. This cooperative heterostructure can trigger gradient recrystallized tribo-layer through plastic deformation co-mediated by dislocation, SFs, and deformation twins (DT) during sliding at RT, thus accommodating elastic–plastic deformation and virtually eliminating wear. The intrinsic composition undulations can stabilize the friction interface and act twofold, not only by circumventing strain localization in the NC tribo-layer but also by densifying the glaze layer. We realize the above strategy in a Ni_50_(AlNbTiV)_50_ (at.%) CCA and reveal the origin of the nanohierarchical architecture by comparing it with its counterpart Ni_75_(AlNbTiV)_25_ CCA with lower alloying concentration (hereinafter named NiX and Ni_3_X, respectively). The wear mechanisms addressed in this work provide guidance for the fabrication of ultra-wear-resistant alloys by exploiting the multicomponent heterostructures in the alloy to activate adaptive friction interface protection during the wide-temperature-range friction process.

## Results and Discussion

### Nanohierarchical architecture with composition undulation

**Fig. 1.**
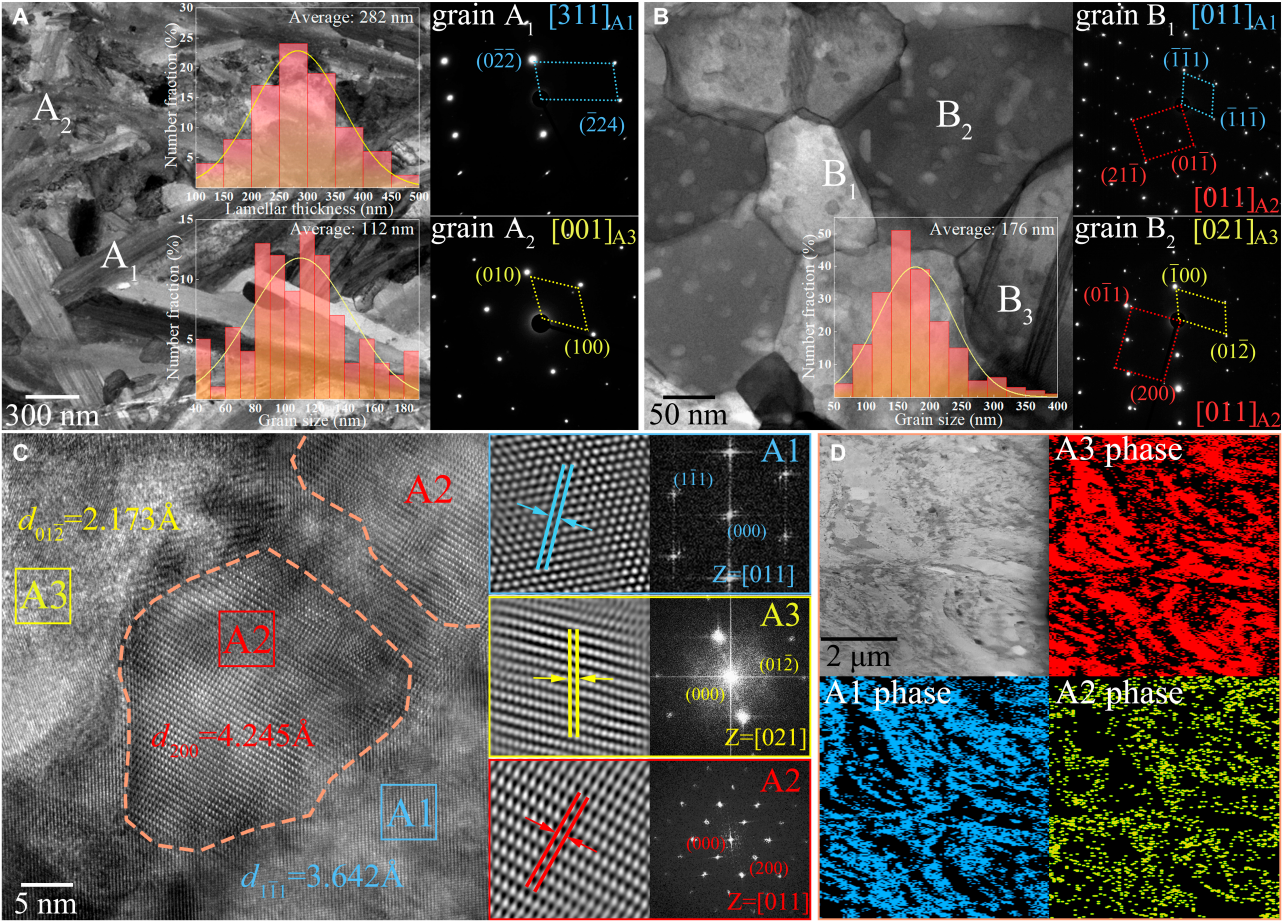
Phase constituent and microstructure in Ni_3_X and NiX. BF-TEM images and SAED patterns showing the grain structures of (A) Ni_3_X and (B) NiX, where the insets are the statistical lamellar thickness or grain size distribution. (C) HRTEM image of the A1 to A3 nano-coupled grains and A2 nanoprecipitates in NiX. Right: IFFT and corresponding FFT images of the marked box regions in the three grains. (D) HAADF-STEM image and corresponding phase mappings showing the separate phases of NiX.

We fabricate the bulk Ni_3_X and NiX by mechanical alloying (MA) followed by molding via spark plasma sintering (SPS). The bright-field transmission electron microscopy (BF-TEM) images in Fig. 1A and B reveal the phase constituents and microstructures in both CCAs. The bulk Ni_3_X has a typical bimodal structure consisting of coarse A1 (disordered face-centered cubic structure) lamellar phases (~74 vol.%) with an average thickness of 282 nm and fine A3 (hexagonal close-packed structure) equiaxed phases (~26 vol.%) with an average size of 112 nm (Fig. 1A and Fig. S1a). By contrast, NiX matrix contains nano-coupled equiaxed grains with an average size of 176 nm (Fig. 1B and Fig. S1b). Moreover, the hierarchical nanoprecipitates are present throughout the interior of the nano-coupled grains, with an average diameter of 20 nm. The selected-area electron diffraction (SAED) patterns prove that the nano-coupled equiaxed grains reflect A1 (such as grain B_1_ in Fig. 1B) and A3 structures (such as grain B_2_ in Fig. 1B), respectively, while the nanoprecipitates have an A2 (disordered body-centered cubic structure) structure. Figure 1C shows a high-resolution TEM (HRTEM) image of the A1 to A3 nano-coupled grains containing two A2 nanoprecipitates. The A2 nanoprecipitates grow in the A1 to A3 nano-coupled grains and create the semi-coherent interfaces with cube-on-cube orientation relationship. Besides, the interfaces of both nano-coupled grains and nanoprecipitates are also free of perceptible micro-defects such as interfacial discontinuities and microgaps/cracks. Statistical content measurements from high-angle annular dark-field (HAADF) scanning transmission electron microscopy (STEM) images and corresponding phase mappings in Fig. 1D show that the estimated volume fractions of A1, A3, and A2 phases are 40 vol.%, 39 vol.%, and 21 vol.%, respectively, and A2 phases are preferentially nucleated in A1 phases. Additionally, there are annealing nano-twins in some of the primary A1 grains in NiX, such as grain B_3_ in Fig. 1B. Figure S2 also shows the presence of annealing nano-twins in the additional A1 grains.

**Fig. 2.**
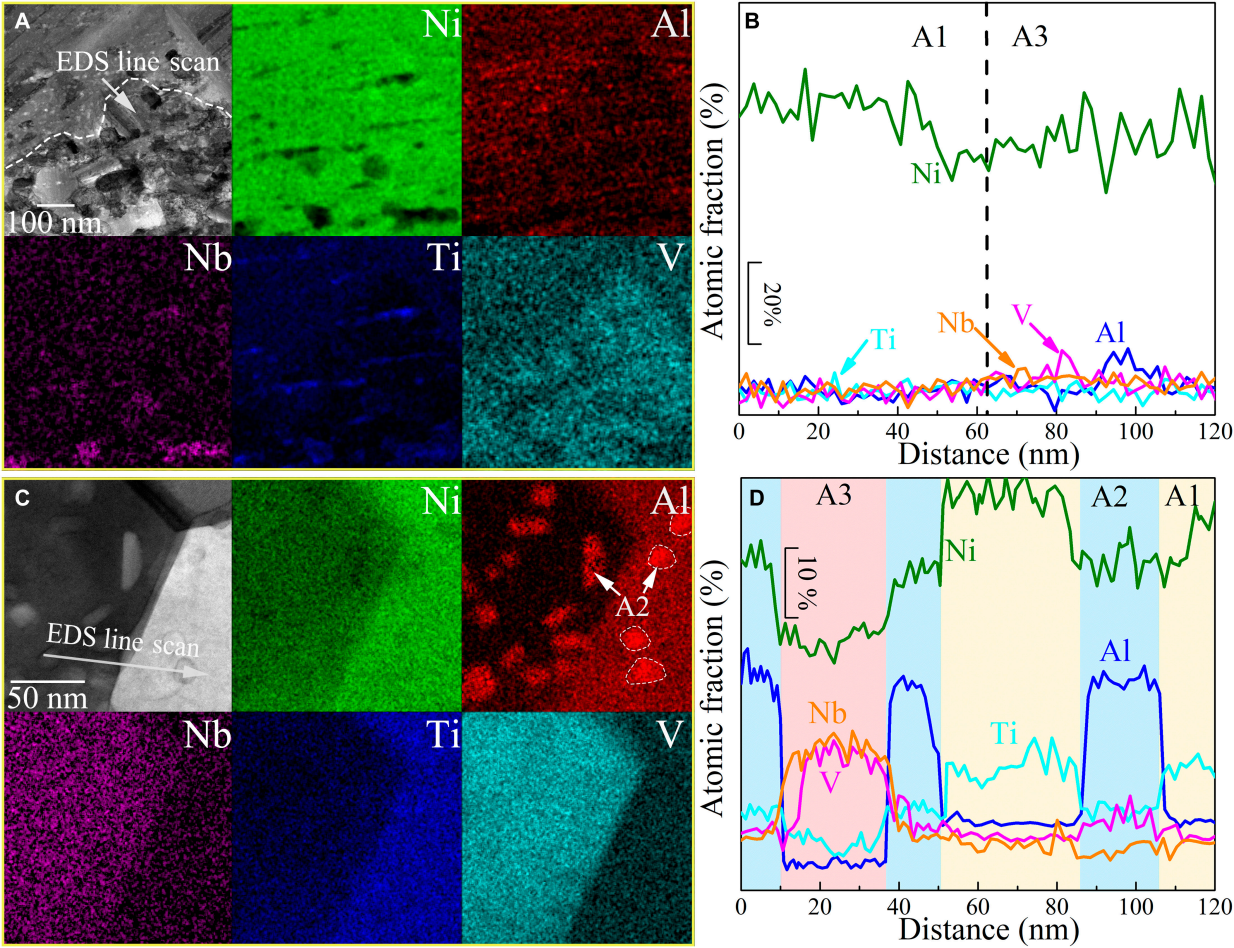
Chemical compositions of each constituent phase in the Ni_3_X and NiX. BF-TEM image and corresponding EDS phase mapping of (A) Ni_3_X and (C) NiX matrix. (B and D) Compositional undulation across the heterophases obtained from EDS line scan marked in panels (A) and (C).

The chemical compositions of the individual constituent phases in the tested CCAs obtained by energy dispersive spectroscopy (EDS) in STEM are shown in Fig. 2 and Table S1. For Ni_3_X (Fig. 2A), the A3 equiaxed phases are slightly rich in Nb and V, whereas other elements have no obvious segregation. In addition, small amounts of (Al, O)-rich and (Nb, Ti)-rich precipitates are distributed in the Ni_3_X matrix, with grain sizes ranging from tens of nanometers to more than 100 nm. The EDS results and SAED patterns in Fig. S3 further reveal the chemical composition and crystal structure of these two types of precipitates. The (Al, O)-rich and (Nb, Ti)-rich precipitates have Al_2_O_3_-type and α-Ti-type close-packed hexagonal structures, respectively. Owing to the low alloying concentration, no pronounced compositional undulations are developed between the heterophases of the Ni_3_X (Fig. 2B). The NiX with high alloying concentration exhibits a unique scenario. Specifically, (Ni, Ti, Al) and (Nb, V) elements appear to enter into the A1 and A3 phases, respectively, whereas Al elements are enriched in the A2 nanoprecipitates (Fig. 2C). The STEM-EDS semi-quantitative analysis indicates that the A1 phase is a Ni-(Ti, Al_0.5_)-based solid-solution phase with some Nb and V, the A3 phase is a (Nb, V)-rich phase, and the A2 nanoprecipitate is a (Ni, Al_0.8_)-based solid-solution phase. Furthermore, there is no additional precipitate or phase formed in the nanohierarchical architecture of NiX (Fig. S4). The spatial distribution of A1 to A3 nano-coupled grains and A2 nanoprecipitates induces inhomogeneous composition distribution in the NiX matrix. Figure 2D shows the line profiles of atomic fraction of individual elements corresponding to EDS mapping, proving the spatial variation of the elemental concentrations throughout the heterophases. Each element has a spatially variable distribution; for instance, Ni fluctuates in the atomic fraction of A1 and A2 phase by up to 63%. The atomic percentages of Ni in the A1, A3, and A2 phases are approximately 58 at.%, 48 at.%, and 38 at.%, respectively. Also, the length of such composition undulations is obviously limited by the spacing between the heterogeneous phases, and the smallest spacing is close to 15 nm. As such, the desired nanohierarchical architecture with composition undulation consisting of A1 to A3 nano-coupled grains and A2 nanoprecipitates is formed in the NiX. It should be emphasized that in both alloys, (Nb, V) are enriched in the A3 phase, whereas in NiX, (Ni, Al, Ti) are enriched in the A1 phase. These elemental segregation phenomena can be attributed to three aspects. First, the binary phase diagrams of these five elements indicate that (a) Nb and V belong to the isomorphous system, which displays complete solubility in liquid (or >300 K) and solid state, and (b) Nb cannot form complete solid solutions with Ti and Ni. Second, the segregation of the solid solution may be influenced by the melting points of the elements. In general, elements with widely differing melting points separate from each other [33,34]. Since both Nb and V possess melting points above 2,100 K, they are rejected by Ni, Al, and Ti. Third, the aggregation of Ni, Al, and Ti is facilitated by the low enthalpy of mixing (<−22 kJ/mol) between them. Conversely, a positive enthalpy of mixing (2 kJ/mol) between Nb and Ti degrades their mutual solid solubilities. Compared to Ni_3_X, the high alloying concentration allows NiX to exhibit more pronounced multi-elemental segregation, thus prompting composition undulations at the nanoscale.

To sum up, the phase information and chemical distribution of Ni_3_X and NiX are closely related to their alloying concentrations. As the content of alloying elements increases from 25 at.% to 50 at.%, the grain structure for the alloy system switches from a dual phase of (lamellar A1 + equiaxed A3) to a multiphase of (equiaxed A1 + equiaxed A3 + A2 nanoprecipitate), resulting in subsequent compositional fluctuations that are dominated by heterogeneous phases. The XRD patterns in Fig. S5 also verify that the phase structure of the bulk Ni_3_X contains the Ni_3_Al-type A1 and Ni_3_Ti-type A3 phases, while the bulk NiX is composed of the T_2_Ni-type A1, Ni_3_Ti-type A3, and NiAl-type A2 phases. High alloying concentrations amplify the chemical complexity of the local atomic environment (e.g., local differences in electronegativity, valence electron concentration, and atomic size), resulting in the formation of solid solutions with high degrees of freedom between neighboring elements [27,35–37]. In particular, the formation of the nanohierarchical architecture inherent to NiX may originate from the specific multicomponent solutal response exerted by the constituent elements during the MA and SPS processes, which requires more detailed simulations and experiments to confirm.

### Remarkable wear resistance and wear mechanisms

**Fig. 3.**
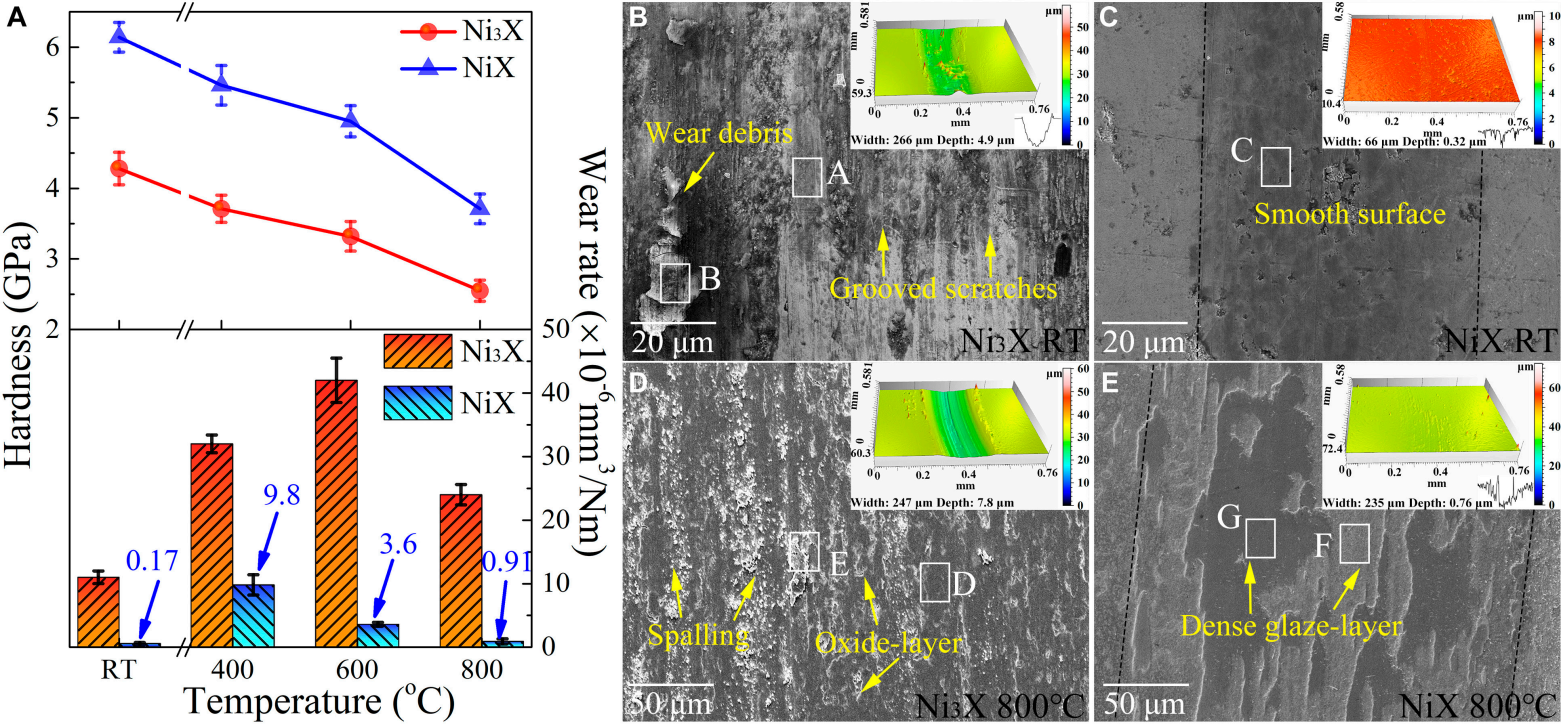
Hardness, wear rate, and worn surface morphology of the tested CCAs. (A) Vickers hardness and wear rate variations of NiX and Ni_3_X in the tested temperature range. (B to E) SEM images of the worn surfaces for Ni_3_X and NiX tested at RT and 800 °C, and chemical compositions of the marked regions are shown in Table S2. Inset: 3D morphologies of worn tracks.

Figure 3A shows the Vickers hardness and wear rate variations of NiX and Ni_3_X as the temperature increases from RT to 800 °C. The hardness of NiX and Ni_3_X gradually decreases from 6.1 GPa and 4.3 GPa at RT to 3.7 GPa and 2.5 GPa at 800 °C, respectively. The high thermal hardness of NiX arises from the solid solution strengthening and the resultant heterostructures [38,39]. The trend in wear rate of the Ni_3_X with temperature is similar to that of the conventional alloys. Specifically, its wear rate increases from 1.1 × 10^−5^ mm^3^/Nm at RT to 4.2 × 10^−5^ mm^3^/Nm at 600 °C, but decreases afterward to 2.4 × 10^−5^ mm^3^/Nm at 800 °C. However, the wear rate of the NiX is basically maintained at the order of (10^−7^ to 10^−6^) mm^3^/Nm over the entire test temperature. In particular, it achieves ultra-low wear at RT (1.7 × 10^−7^ mm^3^/Nm) and 800 °C (9.1 × 10^−7^ mm^3^/Nm). For comparison purposes, the wear rates of the tested CCAs, commercial wear-resistant steels (stainless steel, bearing steel, and low-carbon steel) [40–42], conventional nickel-based alloys (Ni_3_Al/NiAl-based alloys) [20,43–45], high-performance HEAs [3,11,19,21], and bulk self-lubricating composites [17,22,46–48] at different temperatures are summarized in Table S3. It should be noted that the reference materials are compared with our CCAs under similar wear test conditions. Strikingly, we found that the wear rates of NiX are 1 to 3 orders of magnitude lower than those of all the aforementioned comparative materials, representing the highest report level of the wear resistance for the bulk alloy system between RT and 800 °C. Besides, the variations of the coefficient of friction (COF) of the tested CCAs with increasing temperature from RT to 800 °C are similar to the variations of their wear rates (Fig. S6). Specifically, the COF of the Ni_3_X is 0.54 at RT, increasing to about 0.64 between 400 °C and 600 °C, and then decreasing to 0.49 at 800 °C. For NiX, its COF is around 0.25 at RT, increasing to 0.52 at 400 °C, whereas it decreases to 0.43 and 0.36 at 600 °C and 800 °C, respectively.

Figure 3B to E and Fig. S7 show the scanning electron microscope (SEM) images and 3-dimensional (3D) morphologies of the worn tracks for the NiX and Ni_3_X at a temperature range from RT to 800 °C. The results suggest that the wear mechanism of both CCAs between RT and 400 °C is primarily abrasive wear, while their wear between 600 °C and 800 °C is characterized by oxidative and adhesive wear. At RT, Ni_3_X produces a rough worn surface covered with grooved scratches and wear debris as in conventional alloys (Fig. 3B), resulting from strain localization initiated during the groove multiplication process [1,17]. EDS analysis (Table S2) indicates that the wear debris has a higher oxygen concentration compared to the worn surface. Compared with Ni_3_X, the worn surface of NiX avoids grooves and wear debris caused by abrasive wear (Fig. 3C), proving that the sliding-induced elastic–plastic deformation is well accommodated. In particular, the worn track dimensions of the NiX after wear at RT (width of ~66 μm and depth of ~0.32 μm) are close to its surface roughness, proving that abrasive wear is almost eliminated. As is well-known, the *H*/*E* and *H*^3^/*E*^2^ ratios play an essential role in evaluating the elastic and plastic deformability of sliding surfaces during dynamic friction loading, where *H* is the microhardness and *E* is the elastic modulus [8]. In general, a high *H*/*E* implies good capability of the materials to elastic deformation, while a high *H*^3^/*E*^2^ describes good resistance of the material surfaces to crack initiation and propagation during plastic deformation. Therefore, a sliding surface/subsurface with both high *H* and low *E* has the desirable anti-wear capability. We compared the above two parameters from the inside (worn surface) and outside (matrix surface) of the worn tracks for the tested CCAs by nanoindentation tests (Fig. S8). After wear at RT, the results show that both *H*/*E* and *H*^3^/*E*^2^ of the worn surface of NiX (0.0548 and 0.0276) are higher than those of Ni_3_X (0.0306 and 0.0054). Also, the *H*/*E* and *H*^3^/*E*^2^ on the inside of the worn tracks for NiX are 7.5% and 50.8% higher than those on the outside of the worn tracks, respectively, whereas the *H*/*E* and *H*^3^/*E*^2^ on the inside of the worn tracks for Ni_3_X are only 6.6% and 25.5% higher than those on the outside of the worn tracks, respectively. This indicates that sliding substantially improves the ability of NiX to resist elastic and plastic deformation. At 400 °C, the worn surface of Ni_3_X (Fig. S7a) reveals the presence of micro-pits and scattered oxide debris, and a significant increase in deep grooved scratches compared to those at RT, signaling aggravated abrasive wear. No micro-pits or debris are observed from the Ni_3_X surface away from the worn track (Fig. S9), confirming that they are indeed generated during sliding. For NiX, its worn track dimensions also increase at 400 °C compared to those at RT, but the worn surface remains relatively smooth and is free of debris and micro-pits (Fig. S7b). This uncovers that NiX still outperforms Ni_3_X in resisting the elastic–plastic deformation involved with abrasive wear even at 400 °C. The higher high-temperature hardness of NiX may be responsible for the low wear rate compared to Ni_3_X at 400 °C (Fig. 3A). Nanoindentation tests (Fig. S2) also show that *H* on the worn surface of NiX (5.9 GPa) at 400 °C is higher than that of Ni_3_X (4.9 GPa). Combined with the SEM results and EDS analysis, it can be seen that the worn surfaces of both CCAs have a low oxygen concentration (<20 at.%) at 400 °C and do not form a visible protective oxide layer, as evidenced by cross-sectional observations of the worn tracks (Fig. S10a and b). This result is further supported by the fact that the two CCAs have comparable *H* on the inside and outside of the worn tracks. Thus, both alloys exhibit higher wear at 400 °C, which should be ascribed to the softening of the sliding surface and the absence of the protective tribo-layer, compared to RT wear testing.

EDS analysis (Table S2) reveals that the oxygen concentration on the worn surfaces of both tested CCAs increases with increasing test temperature, proving that tribo-oxidation behavior becomes prevalent upon high-temperature wear. In this case, the worn surfaces of the Ni_3_X at 600 °C and 800 °C can be divided into an adhesive zone characterized by plastic spalling and an oxidative zone characterized by local damaged oxide layer (Fig. S7c and Fig. 3D). This indicates that the oxide layers formed by the tribo-oxidation of Ni_3_X are mechanically unstable and struggle to provide good wear protection for the sliding surfaces. Cross-sectional observations of the worn tracks (Fig. S10c and d) further support that the oxide layers of the Ni_3_X are loose and tend to be delaminated, which, in turn, aggravates the spalling and delamination of the tribo-layer. Nanoindentation analysis (Fig. S11) shows that the *H* on the spalling region (2.8 to 3.4 GPa) is obviously lower than that of the local oxide layer (4.3 to 4.4 GPa), possibly making the tribo-layer susceptible to mechanical degradation and failure due to inhomogeneous plastic deformation upon high-temperature wear. In contrast, the worn surfaces of NiX at elevated temperatures are covered by a relatively dense glaze layer (Fig. S7d and Fig. 3E), which matches the cross-sectional observation of the worn tracks (Fig. S10e and f). The glaze layer minimizes the high-temperature wear of the NiX; for example, the depth of worn tracks at 800 °C for NiX is only one-tenth that of Ni_3_X. However, there are differences in the morphology and coverage of glaze layer for NiX at different temperatures. At 600 °C, the glaze layer of NiX is discontinuous, and a fresh substrate surface with low oxygen concentration is exposed at its discontinuities, such as that shown in the region F marked in Fig. S7d. Nanoindentation analysis (Fig. S12) shows that the glaze layers (7.1 GPa) have higher *H* than that at their discontinuities (5.1 GPa). Relatively, the oxygen concentration on the entire worn surface of NiX at 800 °C is above 35 at.% (Table S2), meaning that the worn track is completely covered by a glaze layer. The representative regions F and G in Fig. 3E reflect the glaze layer with bright contrast and the glaze layer with dark contrast, respectively, which arise from the uneven thickness distribution of the glaze layer. The cross-section observation of the worn track (Fig. S10f) confirms the uneven thickness of the glaze layer of NiX at 800 °C. The nanoindentation results (Fig. S12) show that the *H* of the thick glaze layer (7.0 GPa) is similar to that of the thin glaze layer (6.7 GPa), attesting that the worn surface of NiX at 800 °C is completely covered by the glaze layer. The greater the coverage of the glaze layer, the more it can suppress the direct contact between tribo-pairs, thus enhancing the wear resistance of the tribo-system. As a result, the worn track of NiX at 800 °C is only half as deep as that at 600 °C. In other words, the stability and coverage of the glaze layer formed on the worn surface of NiX gradually increases with increasing temperature, favoring a further reduction in wear. In addition, the surface of the Si_3_N_4_ counterbody sliding against Ni_3_X shows an obvious transfer layer, but that of sliding against NiX broadly eliminates the adhesive transfer (Fig. S13). These gentle high-temperature wear behaviors arising from NiX are difficult to attain in conventional alloys.

### Adaptive friction interface protection

**Fig. 4.**
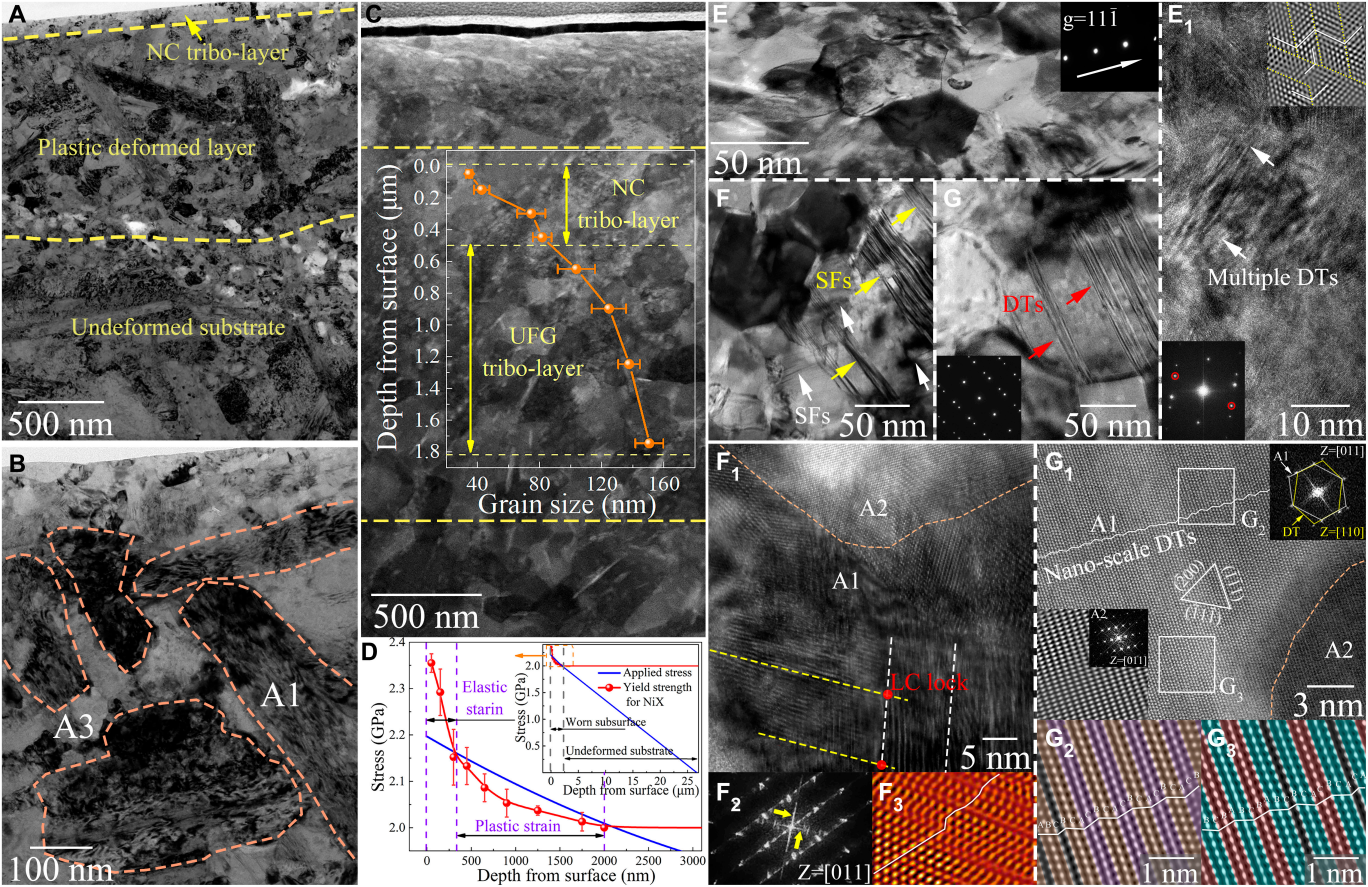
Subsurface microstructure of Ni_3_X and NiX after wear testing at RT. (A and B) BF-TEM image of wear-induced subsurface layer of Ni_3_X. (C) BF-TEM image of the wear-induced subsurface layer of NiX. Inset: Variation of the average grain sizes along the depth direction. (D) Schematic variations of applied stress and yield strength along the depth direction from the worn surface of NiX (see Notes S1 and S2 for detailed calculations). Inset: Variation of the yield strength for NiX with decreasing applied stress to zero. BF-TEM images extending to (E) ~200 nm, (F) ~700 nm, and (G) ~1.6 μm along the depth direction of the top worn surface of NiX, respectively. (E_1_) HRTEM image and its corresponding FFT/IFFT revealing the higher-order multiple nano-scale DTs inside the grains. (F_1_ to F_3_) HRTEM image and corresponding FFT/IFFT showing the LC locks that formed by the reaction of two partial dislocations at different intersecting slip systems. (G_1_ to G_3_) Atomic-resolution HAADF-STEM image and corresponding FFT/IFFT presenting a high density of nano-DTs around the A2 phase, where DT and SF are highlighted by colored and black atomic planes, respectively.

The remarkable wear resistance of the NiX, especially at RT and 800 °C, essentially results from the tuning of the friction interface by the nanohierarchical architecture with composition undulations during sliding. To shed further light on the wear-induced microstructure evolution and tribo-reaction of the tested CCAs, detailed TEM analysis of the worn subsurface layer is performed. At RT, the BF-TEM image (Fig. 4A and Fig. S14a) reveals that the worn Ni_3_X retains a subsurface layer consisting of an extremely thin NC tribo-layer (grain size < 100 nm) with a thickness of ~86 nm at the top and a plastic deformed tribo-layer (grain size similar to that of the substrate) with a thickness of ~1.2 μm at the bottom. The large dimensional difference between these two tribo-layers will inevitably trigger a prominent demarcation of the frictional stress due to the lack of a transition layer. The function of the transition layer is to fill the grain size difference between the NC layer and the substrate, thereby creating a gradient nanostructured surface layer that can progressively release stress [1,4,10]. As shown in the BF-TEM image (Fig. 4B) and dark-field (DF) TEM image (Fig. S14b) of the worn subsurface layer, dislocations are nucleated in the A1 lamellar phases and A3 equiaxed phases, and consequently refine the grains by forming subgrain boundaries. The bimodal/graded grain size distribution causes Ni_3_X surface to undergo heterogeneous deformation during sliding, in which the coarse lamellar A1 grains undergo plastic deformation earlier than the fine equiaxed A3 grains, thereby accumulating a large number of dislocations in the A1 phases [49]. As a result, the dislocation density in A1 phase is higher than that in A3 phase. The refined grains are collected in the top tribo-layer and subsequently cleaved into wear debris during the next groove multiplication process (Fig. 3A) because nanograins are susceptible to local strain [2,4]. The absence of the transition layer causes stress concentrations in the NC tribo-layer that cannot be relieved, which makes the Ni_3_X undergo abrasive wear similar to that of conventional alloys. Instead, the BF-TEM image of worn subsurface for NiX after wear at RT (Fig. 4C) show that two depth-dependent recrystallization regions are evolved underneath the worn surface: a thin NC tribo-layer (with grain size < 100 nm) with a depth extending to ~500 nm from the worn surface; and a middle deformed ultra-fine grained (UFG) tribo-layer (with grain diameter in the range of 100 to 200 nm) with a thickness of ~1.5 μm. The gradient grain refinement that occurs in the subsurface structure against sliding-induced deformation can be observed from the statistical grain size profiles along the depth direction (inset in Fig. 4C). The deformed grains recover to their initial state with increasing depth to ~2 μm. According to Hertzian contact calculations [8,24], a maximum applied stress of 2.2 GPa is imposed on the top worn surface, which exceeds the elastic limit (yield strength) of the NiX (see Note S1 for more details). The maximum penetration depth of applied stress is about 26.7 μm. Meanwhile, we estimate the contribution of gradient grain refinement to the yield strength of subsurface layer based on the Hall–Petch relation [29,50,51], and plot it together with the applied stress in Fig. 4D (see Note S2 for more details). Apparently, the elastic-strain regime and plastic-strain regime corresponding to NC tribo-layer and UFG tribo-layer, respectively, are successively distributed in the recrystallized subsurface layer. The grains underneath the recrystallized tribo-layer (depth > 2 μm) assume an elastically homogeneous stress flow behavior. These phenomena imply that the elastic–plastic deformation derived from the applied stress is well accommodated within the gradient recrystallized tribo-layer and no sharp strain demarcations are generated. Thus, the strain localization of the top tribo-layer is effectively released, which inhibits the initiation of the grooves and debris because the plastic deformation is not trapped on the top surface.

For Ni_3_X, it is difficult to form a transition layer that can relieve stress concentration beneath the NC tribo-layer by relying only on the dislocation reaction in the coarse A1 and fine A3 phases. In stark contrast, the intrinsic heterostructures in the NiX allow the activation of multiple depth-dependent deformation mechanisms upon wear, which promotes the formation of gradient recrystallized tribo-layer. Furthermore, when the grain size is reduced to the nanometer scale, the unusual interface-dependent deformation processes may be triggered [9,52]. Figure 4E to G shows the deformation microstructures extending from the depth direction of the worn surface to 200 nm (NC tribo-layer), 700 nm (top of the UFG tribo-layer), and 1.6 μm (bottom of the UFG tribo-layer), respectively. It can be observed that the dislocation density gradually decreases with increasing depth of the tribo-layer. The dislocation densities of these three regions are determined to be 2.7 × 10^11^ mm^−2^, 6.4 × 10^10^ mm^−2^, and 3.0 × 10^9^ mm^−2^, respectively, by measuring over 10 different grains based on the line-intercept method [9]. It has been reported that heterostructured materials obtain excellent work hardening through hetero-deformation to be compatible with the dynamic loading, owing to the presence of a back stress [49]. Likewise, during the initial stage of sliding, A1 to A3 nano-coupled grains in the top surface rapidly trigger a deformation incompatibility due to the graded distribution of strain. Specifically, the relatively brittle A3 and A2 grains still remain deforming elastically while the soft A1 grains may begin to deform plastically. Then, to adapt these hetero-deformation near phase/grain interfaces and alleviate the consequent distortion energy, geometrically necessary dislocations (GNDs) are required. Generally, accumulated GNDs can induce strain partitioning and directly boost two types of hardening mechanisms [53]: kinematic hardening by directional back stress at grain boundaries (GBs) of soft A1 grains and isotropic hardening by cross-slip of the forest dislocations inside the nano-coupled grains. As such, the grain structure in the NC tribo-layer (Fig. 4E) suggests that many dislocations block each other and locally raise the energy, which, in turn, drives their own reorganization into sub/low-angle GB and accelerates new grain growth [2]. Some dislocation-free ultrafine grains provide additional evidence for dynamic recrystallization, as new grains consume existing dislocations in this region prior to their nucleation. Moreover, HRTEM image and its corresponding FFT/IFFT inside the grains (Fig. 4E_1_) indicate that higher-order multiple DTs with polarity are activated. Nano-scale twin boundaries (TBs) refine the microstructure into an alternating laminar twin-matrix structure by providing high-angle GBs in the parent grains [13]. Thus, the significant grain refinement in the NC tribo-layer benefits from the interaction of dislocations and nano-scale DTs during plastic deformation. Long-range back stress can accumulate dislocations at the interface while constantly strengthening the A1 grains. HRTEM image and its corresponding IFFT (Fig. S15) show that the atomic arrangement in the vicinity of GB for nano-coupled grains is quite different from that before wear. There are a lot of distortions and misfit dislocations at the interface, which can act as accessional edge-pinning points to hinder dislocation climbing and slipping. These dislocations that accumulate at the semi-coherent interfaces of the nano-coupled grains further strengthen the NC tribo-layer. Thus, actual strength increment obtained for the NC tribo-layer should exceed the results shown in the Fig. 4D. It is worth mentioning that the NC tribo-layer in the NiX does not suffer from collapse-like localized fracture and delamination as in other nanograined alloys, which is attributed to the spatial composition undulation in the matrix increasing the strain-rate sensitivity and causing sluggish dislocation motion due to dislocation interlocking, thereby promoting the dislocation storage capacity of the friction interface during sliding [54]. As the depth extends to the UFG tribo-layer, multiple SFs and DF-mediated deformation modes are observed (Fig. 4F and G), albeit with a decrease in dislocation density. Concretely, a widespread class of parallel SF bundles, with an average spacing of 10 nm, were observed in the topmost A1 grain of the UFG tribo-layer (Fig. 4F), which decreases in the spatial density with increasing the depth. Numerous SFs belong to {111}_A1_ type across the nano-coupled grain and intersect with some short SFs along another {111}_A1_ slip system to form unique nano-spaced SF networks. HRTEM observations (Fig. 4F_1_) and Fig. S16a) reveal significant storage and interaction of Shockley partial dislocations near the A2 phases. The A2 phases mediate the plastic deformation in the UFG tribo-layer through the interlaced SF network around them, which is distinct from the deformation mechanism in the NC tribo-layer. Concurrently, an immobile stair-rod dislocation along the non-slipping plane, the so-called the sessile Lomer-Cottrell (LC) locks, is initiated by the intersection between the two leading partial dislocations [29], as shown in Fig. 4F_1_ and Fig. S16b. Besides, the crossing diffraction fringes in the FFT pattern of the [011] zone axis (Fig. 4F_2_ and Fig. S16b) and the representative stacking sequence in its IFFT (Fig. 4F_3_) further verify the existence of intersected SFs. These SF networks and LC locks can not only refine the microstructure but also evolve into Frank-Read sources that motivate dislocation multiplication, achieving a progressive and stead work hardening in the UFG tribo-layer of the NiX [54,55]. As the depth increases to the bottom of the UFG tribo-layer (Fig. 4G), the influence of multiple SFs on the microstructure gradually weakens. However, there are large numbers of parallel lamellae detected in this region. The main components of these lamellae are DTs, as evidenced by the SAED (Fig. 4G). Close-up atomic-resolution HAADF-STEM view (Fig. 4G_1_) and IFFT (Fig. 4G_2_ and G_3_) reveal that each individual long lamellae interface consists essentially of a high density of nanoscale DTs with a few SFs, which are formed along the {111}_A1_ habit plane. Measurements of the thickness between adjacent TBs show an extremely smaller value, viz., below 1 nm on average, corresponding to a super high average twin density (~2 × 10^9^ m^-1^) in the lamellae. The thickness of these DTs is quite small, even below the supra-nano range (1 to 10 nm) [56]. These coherent TBs obey the Burgers orientation relationship with the hierarchical A2 phase, by which the direction of TB and close-packed plane of A2 phase are parallel. The high density of nanoscale DT subdivides the microstructure around the A2 phase into a sub-1-nm-thick twin/matrix lamellar nanostructure. The densely stacked TB arrays are able to substantially hinder dislocation motion, providing increased strength while improving thermal/mechanical stability through the release of internal stress and strain energy [30,57]. Also, they can serve as extra independent shear carriers to further offer additional kinematic degrees of freedom for dislocation transmission inside the nanotwinned regions, effectively enhancing the plastic deformability of the material [56,58]. From the above, we rationalize that the extremely high density of SFs and DT/TB-mediated plastic deformation are mainly responsible for the excellent wear resistance and load-bearing capacity of the UFG tribo-layer. The formation of both multiple SFs and nanoscale DTs is associated with the competition between the slip of full dislocations and the emission of partial dislocations [30]. More intriguingly, the hierarchical A2 nanoprecipitates play a pivotal role in activating the interplay of partial dislocations in the UFG tribo-layer. The following four factors contribute to the propensity for extensive SF or DT interactions in the vicinity of the A2 phase. First of all, the high probability for partial dislocations and SFs/DTs nucleation may stem from the complex chemical features and hierarchical architecture inherent to NiX, in which the saliently rugged local atomic environments associated with low stacking fault energy (SFE) are created at the nanoscale. One of the effective strategies to activate SF and DT during deformation is to reduce the SFE of the system by tuning the composition [30]. According to classical Orowan relation [59], as the SFE decreases, the partial dislocation activity becomes more favored, while the full dislocation activity is substantially suppressed. The small regions with reduced SFE may form around the A2 nanoprecipitates due to the depletion of high-SFE elements (Ni and Al) [55]. Second, the dislocations cut through the hetero-interface between A1 and A3 phases (Fig. S15), whereas they are pinned by the A2 nanoprecipitates (Fig. S16). These hierarchical A2 phases dramatically strengthen the friction interface through dislocation bypassing, resulting in high strain hardening, which, in turn, interrupts the local strain softening and damage initiation caused by the shearable matrix phase [5,50]. At higher sliding-induced deformations, high stress level, which arises from the complex interactions between dislocations and nanoprecipitates, is capable of activating DTs even in the A1 phase with high SFE [60]. The ensuing formation of nano-DTs allows for the continuous accumulation of dislocations as sustainable sources of high-density dislocation storage, further hardening and toughening the worn subsurface layer [10,56]. Third, incompatible plastic deformation in the heterostructure consisting of A1 to A3 nano-coupled grains and hierarchical A2 nanoprecipitates is accompanied by a graded stress/strain state with the uneven strain partitioning and the presence of the back stress, especially at the early stage of sliding-induced plastic deformation [49]. Microscopically, the piling up of long-range back stresses around the A2 phases tend to trigger more GNDs and stronger local stress fields, which boost the lattice distortion energy [30,53]. This endows the internal driving force for partial dislocations to slip out of equilibrium separation while progressively retaining dense SFs/DTs [30,54]. Last but not least, previous studies have demonstrated that local stress concentrations during plastic deformation are expected to increase the probability of partial dislocation activity [61]. It is trivial to instigate localized stress concentrations during the hetero-deformation controlled by the current nano-coupled grains. As the example in the Fig. S17a and b, sparse DTs are observed in certain regions, proving that the local stress in these regions reaches the critical resolved shear stress required for twin nucleation [58]. Taken together, the intrinsic heterostructures of the NiX have an appreciable potential to activate partial dislocations and the resultant SF/DT interactions during sliding-induced plastic deformation. Apart from the gradient recrystallized tribo-layer, the surface oxides formed during wear also affect the wear resistance of NiX. Since we incorporated a high concentration of oxidation-prone elements in the NiX, some discontinuous nano-scale oxide layers are formed on the top surface (Fig. S17c and d), which may be considered as dispersed reinforcing phases to further suppress the deformation. All intrinsic heterostructures in NiX, including A1 to A3 nano-coupled grains and hierarchical A2 nanoprecipitates, jointly mediate frictional loading, creating the spatial playground for triggering a strong NC tribo-layer and the resultant formation of a deformable UFG tribo-layer to accommodate elastic–plastic deformation. This competition and coexistence of multiple deformation pathways along the depth direction of the frictional stress is beneficial to gradually release the stress concentrations at the friction interface and thus eliminates wear. The mentioned strengthening mechanisms are also the fundamental reason for the high *H*, *H*/*E*, and *H*^3^/*E*^2^ obtained on the worn surface of NiX during sliding.

**Fig. 5.**
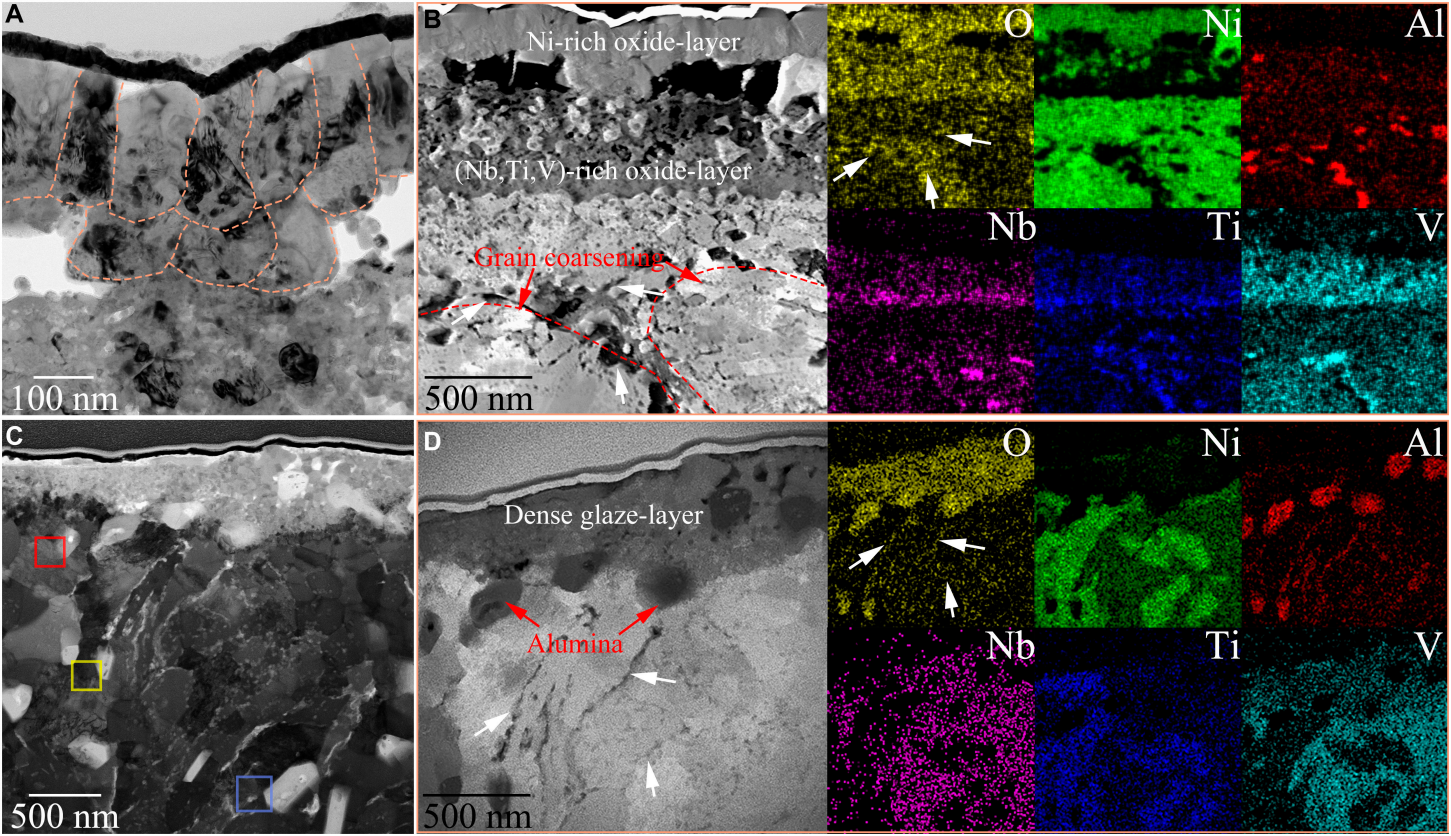
Subsurface microstructure of Ni_3_X and NiX after wear testing at 800 °C. (A) BF-TEM image of the oxide layer of Ni_3_X. (B) HAADF-STEM image and corresponding EDS mapping of the subsurface layer of Ni_3_X. (C) BF-TEM image of the worn subsurface layer of NiX. (D) HAADF-STEM image and corresponding EDS mapping of the subsurface layer of NiX. White arrows represent the nanoscale O-rich regions, reflecting the inward diffusion of the oxygen anions.

During wear at elevated temperatures, the oxide layer formed on the two CCAs dominated their resistance to adhesive and oxidative wear. The loose oxide layer increases the wear of the Ni_3_X, while the dense glaze layer imparts ultra-low wear to the NiX. The BF-TEM image in Fig. 5A proves that the oxide layer of the Ni_3_X at 800 °C consists of an outer columnar crystal (with a diameter of ~120 nm) oxide layer with a thickness of ~317 nm and an internal nanocrystal oxide layer with a thickness of ~475 nm. The HAADF-STEM image and the corresponding EDS mapping in Fig. 5B show that the outer oxide layer and the internal oxide layer of the Ni_3_X are enriched with Ni and (Nb, Ti, V) elements, respectively. SAED patterns (Fig. S18a) confirm that the outer oxide layer consists of NiO columnar crystals while the internal oxide layer contains Nb_2_O_5_, TiO_2_, and V_2_O_5_ nanocrystals. The presence of visible gaps at the interface between the two oxide layers suggests that they are prone to separate from each other during sliding, thereby exacerbating delamination and spalling of the tribo-layer. Besides, the white arrows in Fig. 5B indicate that the environmental oxygen flows inward along the worn subsurface layer and promotes the formation of (Al, O)-rich nanoparticles with an average grain size of 64 nm. Underneath the oxide layer, coarsening of A1 lamellar grains and A3 equiaxed grains occurs, with an increase in average thickness/size to about 340 nm and 152 nm, respectively, in a depth span of 1 μm to 3.5 μm. High-temperature sliding reduces the energy barrier for GB sliding/rotation and then mechanically drives grain coarsening in the subsurface layer [4,5]. This coarsening behavior that occurs in the subsurface layer also degrades the wear resistance of the Ni_3_X. For NiX, the protective glaze layer that grows densely on the worn surface dramatically minimizes oxidative and adhesive wear between the tribo-pairs. BF-TEM images in Fig. 5C and Fig. S18b show that the subsurface layer of the NiX at 800 °C consists of a glaze layer with an average thickness of 393 nm and a deformed tribo-layer in a depth span of 0.5 μm to 3 μm. EDS mapping (Fig. 5D) shows that the glaze layer is enriched in Nb, Ti, and V elements. SAED pattern (Fig. S18b) demonstrates that the glaze layer comprises nanocrystalline metallic oxides including Nb_2_O_5_, TiO_2_, V_2_O_5_, and NiO. EDS mapping (Fig. 5D) and HRTEM image (Fig. S19) confirm the formation of some (Al, O)-rich nanoparticles (Al_2_O_3_) with an average grain size of 175 nm along the glaze layer and the deformed tribo-layer. Furthermore, the glaze layer of the NiX is dense and has a good metallurgical bond with the substrate, which is a prerequisite for the excellent wear resistance of the oxide layer. The formation mechanism of oxide/glaze layer on the worn Ni_3_X and NiX can be interpreted both thermodynamically and kinetically. In the current CCA system, the formation enthalpies for the oxides of the alloying elements Al (∆*H*_Al_2_O_3__ =  −1,675.7 kJ/mol), Nb (∆*H*_Nb_2_O_5__ =  −1,899.5 kJ/mol), Ti (∆*H*_TiO_2__ =  −944 kJ/mol), and V (∆*H*_V_2_O_5__ =  −1,551 kJ/mol) are significantly lower than that of the matrix element Ni (∆*H*_NiO_ =  −240.6 kJ/mol). Hence, the thermodynamic oxidation order of the constituent elements of Ni_3_X and NiX is Nb → Al → V → Ti → Ni. However, the actual oxidation process is also governed by kinetic ion transportation, which is related to the solute concentrations under the constant oxidation temperature and time [21,62]. For Ni_3_X, the oxides of Ni become competitive during tribo-oxidation due to the concentration of Ni up to 75 at.%. This allows the preferential formation of NiO-dominated oxide layer on the worn surface of Ni_3_X and triggers the following oxidation kinetic behaviors. First, the faster formation of Ni-oxides can deplete more Ni ions/atoms in the subsurface layer beneath the outer NiO layer and thus amplify the concentration gradients of this solute atom, which will contribute to its atomic diffusion to the sliding surface. The relative decrease in the concentration of the matrix element (Ni) favors the generation of an interfacial oxide layer consisting of Nb_2_O_5_, TiO_2_, and V_2_O_5_ nanocrystals. Second, Nb/V and Ti are usually switched to penta-valent and tetra-valent cations, respectively, during oxidation. The Nb^5+^, V^5+^, and Ti^4+^ ions dissolved in the NiO layer can act as high-valent dopants to improve the vacancy concentration of the oxide layer, accelerating the interdiffusion of oxygen and metal ions and thus increasing the thickness of the oxide layer [63]. Moreover, the high vacancy concentration tends to initiate the creation of micro-voids in the oxide layer, which makes the oxide layer loose and less protective [64]. Third, the mismatched thermal expansion coefficients between the outer oxide layer and the interfacial oxide layer are bound to induce high thermal stresses at their interfaces, which can exacerbate the spallation of the oxide layer at elevated temperatures [62]. Additionally, since aluminum oxide has a slower growth rate compared to other oxides, only discontinuous (Al, O)-rich nanoparticles are formed in the worn subsurface layer [65]. Instead, NiX contains alloying elements with a concentration comparable to that of the matrix element, which facilitates enhanced oxidation kinetics between the alloying elements and oxygen during the tribo-oxidation. This phenomenon is verified by x-ray photoelectron spectroscopy (XPS) analyses on the worn surfaces of the two CCAs (Fig. S20). Specifically, the relative integrated areas of the various metal oxides (Al_2_O_3_, Nb_2_O_5_, TiO_2_, and V_2_O_5_) in the spectra of Al2p, Nb2p, Ti2p, and V2p increase with increasing concentrations of the alloying elements(from Ni_3_X to NiX). This enables the worn surface of NiX to be preferentially covered by a glaze layer composed of (Nb, Ti, V)-rich nanocrystalline metallic oxides during tribo-oxidation. Likewise, because of the slow growth rate of the aluminum oxide, only discontinuous Al_2_O_3_ nanoparticles are formed. Nevertheless, the size of the (Al, O)-rich nanoparticles formed in the worn subsurface layer of NiX is larger than that in Ni_3_X. Previous studies have shown that the Nb-rich and V-rich glaze layer has excellent anti-wear properties and oxidation resistance during high-temperature wear [25,66]. Meanwhile, the high concentration of oxidizable elements (like Nb and Al) lowers the combined enthalpy between the worn surface and environmental oxygen, accelerating the formation of a protective glaze layer and thus preventing further oxidation of the worn subsurface layer. Similar results on the glaze layer of the Al/Nb-containing alloy system have been found by previous studies [24,25] whereby the glaze layer becomes dense as the concentration of Al/Nb in the matrix increases.

In particular, there are two other wear responses arising from tribo-oxidation penetration of NiX that deserve to be signaled. First, Al_2_O_3_ nanoparticles are precipitated discontinuously throughout the subsurface layer. It is conceivable that the Al-rich A2 nanoprecipitates accelerates the adsorption of oxygen on the worn surface while naturally acting as the active catalysts to offer abundant pathways for the inward diffusion of oxygen anions, which allows the nucleation of alumina. This inference is verified by the presence of numerous nanoscale O-rich regions in the deformed tribo-layer observed from the EDS mapping for O element (white arrows in Fig. 5D). On the basis of the Orowan bypass model, since the elastic modulus of alumina (~375 GPa) is much higher than that of NiX (137 GPa), the stress is bound to rise drastically when it flows to the interface between the alumina nanoparticles and the NiX matrix [67]. This assists in enhancing the deformability of the subsurface layer. Second, the SAED pattern (Fig. S18b) in the deformed tribo-layer reveals the coexistence of several crystalline rings and an amorphous halo ring inside. Several sets of SAED patterns (Fig. S19a to d) and HRTEM images (Fig. S19f and g) confirm that nanometer-sized amorphous oxides are prevalently embedded in the nanocrystal matrix adjacent to the Al_2_O_3_ nanoparticles. Moreover, the tribo-layer regions away from the Al_2_O_3_ nanoparticles and the undeformed substrate regions still maintain the initial structure of NiX (Figs. S18b and S19e), which excludes the possibility of amorphous oxides arising from the intrinsic structure of the alloy or from irradiation/ion bombardment during TEM foil sample preparation. Thus, the formation of amorphous oxides is triggered by the tribo-oxidation behavior along the subsurface layer. It has been shown that dissolving the oxygen anion in the alloy can change the atomic coordination through directional bond contributions, resulting in the in situ formation of amorphous oxides upon wear-induced plastic flow [7,12]. Indeed, the NiX does have good glass-forming ability attributable to the fact that it contains more than three alloying elements with significant atomic size difference and negative enthalpy of mixing. It is known that introducing amorphous in solid solution matrix to form topological nanocomposites is one of the effective ways to promote multiple dislocation activity and thus obtain homogeneous deformation [2,57], as evidenced by the high density of dislocations and SFs congested at the interface of the amorphous oxides (Fig. S21). Nanoindentation analysis (Fig. S22) proves that the *H* and *E* of the deformed tribo-layer (7.6 GPa and 179 GPa) containing alumina nanoparticles and amorphous oxides are higher than those of the undeformed substrate (7.0 GPa and 134 GPa). The deformed nanocomposite tribo-layer synergistically enhances the deformability of the friction interface, thus preventing delamination and spalling of the glaze layer. Moreover, the average grain size of A1 to A3 nano-coupled grains in the worn subsurface layer is measured to be ~195 nm. In comparison with the as-fabricated NiX, such slight increase in grain size reflects the good thermal stability of the NiX during high-temperature wear. The good thermal stability of NiX is attributed to the elevated heterogeneity due to compositional undulations, which may give rise to the multielement cosegregation-driven kinetic stabilization of grains due to the solute-drag effect [39]. Concurrently, the in situ formation of alumina nanoparticles and amorphous oxides further inhibits the grain coarsening by dissipating frictional energy. Therefore, the cooperative heterostructure in the NiX activates the dense nanocrystalline glaze layer during high-temperature wear, thus minimizing the adhesive and oxidative wear between the tribo-pairs.

From the above analyses, the underlying wear mechanisms responsible for the unprecedented wear resistance in the NiX are schematically illustrated in Fig. S23. During sliding at RT, the intrinsic structures of NiX rely on multiple deformation pathways to evoke the NC tribo-layer and the UFG tribo-layer, thus progressively accommodating the gradient elastic–plastic deformation distributed along the friction interface. During sliding at 800 °C, the intrinsic structures of NiX are able to promote the formation of a dense glaze layer and a deformed tribo-layer containing alumina nanoparticles and amorphous oxides through spontaneous tribochemical reactions, which dramatically suppress adhesive and oxidative wear between tribo-pairs.

## Conclusion

To summarize, this work proposes a paradigm for the development of ultra-wear-resistant alloys by combining A1 to A3 nano-coupled grains with A2 nanoprecipitates. The designed NiX is capable of activating favorable microstructure evolution and tribo-reaction during sliding, an effect that we refer to as adaptive friction interface protection. Contributing to the roles co-played by nanohierarchical architecture and spatial composition undulation, bulk NiX achieves ultralow wear rate between RT and 800 °C, which heretofore represents one of the highest wear resistance reported for any bulk alloys or composites. Such wear response found in the current CCA can be selectively applicable to other alloy systems to achieve pleasing wear resistance.

## Materials and Methods

### Material preparation

The bulk Ni_3_X and NiX samples were prepared via high-energy ball milling (FRITSCH, Germany) followed by SPS (LABOX-3010KF). Elemental powders of Ni, Al, Nb, Ti, and V with high purity (99.9%) and particle sizes below 45 μm were used as starting materials for synthesizing MA powders. Ball milling was carried out in a high-purity argon atmosphere and operated intermittently for 32 h at a rotational speed of 300 r/min. The grinding ball was made of cemented carbide and the ball-to-powder weight ratio was set to 5:1. The alloyed powders were consolidated by SPS at 1,050 °C for 8 min under a preset pressure of 35 MPa, the average heating rate was 70 °C/min, and the vacuum pressure was below 5 Pa. The testing specimens were made of the middle section of the SPS samples and the surface layer of the SPS samples was removed by about 1 to 2 mm thick (contamination/reaction layer) before machining.

### Physical, mechanical, and tribological property measurements

The density of the CCAs was determined by a true density analyzer (AccuPyc-1330). The specific heat and thermal conductivity of the CCAs at RT were determined by a laser thermal conductivity testing instrument (S/N:257-1-316, Germany). High-temperature Vickers hardness tests were performed on an HTV-PHS30 hardness tester (Archimedes Industry Technology Co., UK) under a load of 5 N for 10 s. The sample size of the hardness test was 3 × 10 × 10 mm. The nanoindentation tests were performed on the Anton-Paar NHT3 TriboIndenter system with a Berkovich indenter at RT, with a loading rate of 0.1 mN/s and a peak load of 3.0 mN. A commercial ball-on-disc tribometer (HT-1000, Zhongke-Kaihua Co. Ltd) was used to evaluate the wear evolution of the tested CCAs in the temperature range from RT to 800 °C. The sample size for wear test was Ø 20 × 4 mm (roughness of Ra < 0.02 μm). Si_3_N_4_ balls (Ø 6 mm, Vickers hardness of ~15 GPa) were selected as counter materials. Wear tests were conducted at a normal load of 5 N, a sliding speed of 0.2 m/s, and a sliding distance of 360 m. The cross-section profiles and wear volume of the worn tracks were measured by a 3D-optical profilometer (MicroXAM-800). Wear rates of the tested CCAs were calculated as the wear volume divided by applied load and sliding distance. The COF values were automatically recorded by a tribometer. The above tests on physical, mechanical, and tribological properties were repeated at least three times.

### Microstructural characterizations

The crystal structure and phase constituent of the processed samples were examined by x-ray diffraction (XRD-EMPYREAN, Netherlands). Scanning electron microscopy (SEM, Quanta-450FEG) equipped with energy dispersive spectrometry (EDS) and a backscatter electron (BSE) detector was utilized for microstructure identification. The transmission electron microscopy (TEM, JEOL JEM-ARM200) with SAED and EDS was used to obtain the grain structures and phase compositions. HAADF-STEM, corresponding bright-field (BF), dark-field (DF), and high-resolution TEM (HRTEM) images of the specific area were analyzed accordingly. The TEM phase mappings were obtained using Pathfinder software. The volume fraction of each phase was estimated by ImageJ software. The wear mechanism and tribochemical reaction of frictional interfaces were deduced by SEM-EDS and XPS (ESCALAB 250XI). The detailed microstructure evolution and composition distribution of the worn subsurface were characterized using STEM (JEOL JEM-F200) with an attached EDS detector. TEM foil samples were prepared by a focused ion beam system (FEI Helios Nanolab). A protective platinum strip with a thickness of 1 μm was deposited on the worn track to avoid damage by the gallium ion source. The tribochemical reaction of worn surfaces was analyzed by XPS (ESCALAB 250XI).

## Data Availability

All data needed to evaluate the conclusions in the paper are present in the paper and/or the Supplementary Materials. Additional data related to this paper may be requested from the authors.

## Supplementary Materials

Supplementary 1 Fig. S1. BF-TEM images showing the grain structures of (A) Ni_3_X and (B) NiX. Fig. S2. Annealing nano-twins in the primary A1 grain of NiX. Fig. S3. Microstructure and chemical composition of two precipitates in Ni_3_X. Fig. S4. Chemical composition of the nanohierarchical architecture consisting of A1 to A3 nano-coupled grains and A2 nanoprecipitates in NiX. Fig. S5. XRD patterns of the MA powders at different milling durations and the sintered samples. Fig. S6. Coefficient of friction (COF) curves of NiX and Ni_3_X obtained during sliding from RT to 800 °C. Fig. S7. Worn surface morphology of the tested CCAs at 400 °C and 600 °C. Fig. S8. Load–displacement curves of the inside and outside of the worn tracks for the tested CCAs after wear testing at RT and 400 °C. Fig. S9. SEM image of the Ni_3_X surface away from the worn track after wear testing at 400 °C. Fig. S10. Cross-sectional morphologies of worn tracks for the Ni_3_X and NiX after wear testing between 400 °C and 800 °C. Fig. S11. Optical images of the worn surface for the tested Ni_3_X after wear at elevated temperatures, and the load–displacement curves of the corresponding regions marked therein. Fig. S12. Optical images of the worn surface for the tested NiX after wear at elevated temperatures, and the load–displacement curves of the corresponding regions marked therein. Fig. S13. Surface morphology and element distribution the Si_3_N_4_ counterbody sliding against Ni_3_X and NiX tested between 600 °C and 800 °C. Fig. S14. Subsurface microstructure of Ni_3_X after wear testing at RT. Fig. S15. Dislocation interactions at the interface between heterophases in the tribo-layer of NiX. Fig. S16. SF network (dashed line) and Lomer–Cottrell (LC) locks (red circles) around A2 phase in the UFG tribo-layer of NiX. Fig. S17. Top worn subsurface microstructure of NiX after wear testing at RT. Fig. S18. Subsurface layer microstructure of Ni_3_X and NiX after wear testing at 800 °C. Fig. S19. Nanometer-sized amorphous oxides formed near alumina nanoparticles in the subsurface layer of NiX after wear testing at 800 °C. Fig. S20. Chemical states of the worn surfaces for CCAs after wear testing at 800 °C. Fig. S21. HRTEM image of the yellow box marked in Fig. S19a or Fig. 5C. Fig. S22. Load–displacement curves obtained from the cross-section of the worn track of NiX after wear testing at 800 °C. Fig. S23. Schematic diagram illustrating the wear mechanisms associated with adaptive friction interface protection in the NiX during sliding at RT and 800 °C, respectively. Table S1. STEM-EDS measured compositions of each phase in Ni_3_X and NiX. Table S2. Chemical composition (at.%) of the worn surfaces for NiX and Ni_3_X. Table S3. Comparison of wear resistance for the current CCAs with other reported high-performance bulk alloys and composites. Note S1. Estimate of the Hertzian contact stress and flash contact temperature during wear test at RT. Note S2. Theoretical calculations of yield strength increments along the worn subsurface layer of NiX after wear at RT.
