# Supplementary material for: Remarkable Wear Resistance in a Complex Concentrated Alloy with Nanohierarchical Architecture and Composition Undulation
Source: Research (Wash D C). 2023 Jun 5;6:0160. doi: 10.34133/research.0160 (PMC10241162; doi:10.34133/research.0160)
Supplement: Supplementary 1 — Fig. S1. BF-TEM images showing the grain structures of (A) Ni3X and (B) NiX. Fig. S2. Annealing nano-twins in the primary A1 grain of NiX. Fig. S3. Microstructure and chemical composition of two precipitates in Ni3X. Fig. S4. Chemical composition of the nanohierarchical architecture consisting of A1 to A3 nano-coupled grains and A2 nanoprecipitates in NiX. Fig. S5. XRD patterns of the MA powders at different milling durations and the sintered samples. Fig. S6. Coefficient of friction (COF) curves of NiX and Ni3X obtained during sliding from RT to 800 °C. Fig. S7. Worn surface morphology of the tested CCAs at 400 °C and 600 °C. Fig. S8. Load–displacement curves of the inside and outside of the worn tracks for the tested CCAs after wear testing at RT and 400 °C. Fig. S9. SEM image of the Ni3X surface away from the worn track after wear testing at 400 °C. Fig. S10. Cross-sectional morphologies of worn tracks for the Ni3X and NiX after wear testing between 400 °C and 800 °C. Fig. S11. Optical images of the worn surface for the tested Ni3X after wear at elevated temperatures, and the load–displacement curves of the corresponding regions marked therein. Fig. S12. Optical images of the worn surface for the tested NiX after wear at elevated temperatures, and the load–displacement curves of the corresponding regions marked therein. Fig. S13. Surface morphology and element distribution the Si3N4 counterbody sliding against Ni3X and NiX tested between 600 °C and 800 °C. Fig. S14. Subsurface microstructure of Ni3X after wear testing at RT. Fig. S15. Dislocation interactions at the interface between heterophases in the tribo-layer of NiX. Fig. S16. SF network (dashed line) and Lomer–Cottrell (LC) locks (red circles) around A2 phase in the UFG tribo-layer of NiX. Fig. S17. Top worn subsurface microstructure of NiX after wear testing at RT. Fig. S18. Subsurface layer microstructure of Ni3X and NiX after wear testing at 800 °C. Fig. S19. Nanometer-sized amorphous oxides formed near [file research.0160.f1.docx]

**Remarkable Wear Resistance in a Complex Concentrated Alloy with Nanohierarchical Architecture and Composition Undulation**

**Authors**

Yushan Geng^1,2^, Wenyuan Chen^1^, Hui Tan^1^, Jun Cheng^1,2,3^*, Shengyu Zhu^1^, Jun Yang^1,2,3^*, Weimin Liu^1,3^

**Affiliations**

^1^ State Key Laboratory of Solid Lubrication, Lanzhou Institute of Chemical Physics, Chinese Academy of Sciences, Lanzhou 730000, PR China

^2^ Center of Materials Science and Optoelectronics Engineering, University of Chinese Academy of Sciences, Beijing 100049, PR China

^3^ Shandong Laboratory of Yantai Advanced Materials and Green Manufacturing, Yantai 264000, PR China

Correspondence should be addressed to Jun Cheng; chengjun@licp.cas.cn and Jun Yang; jyang@licp.cas.cn


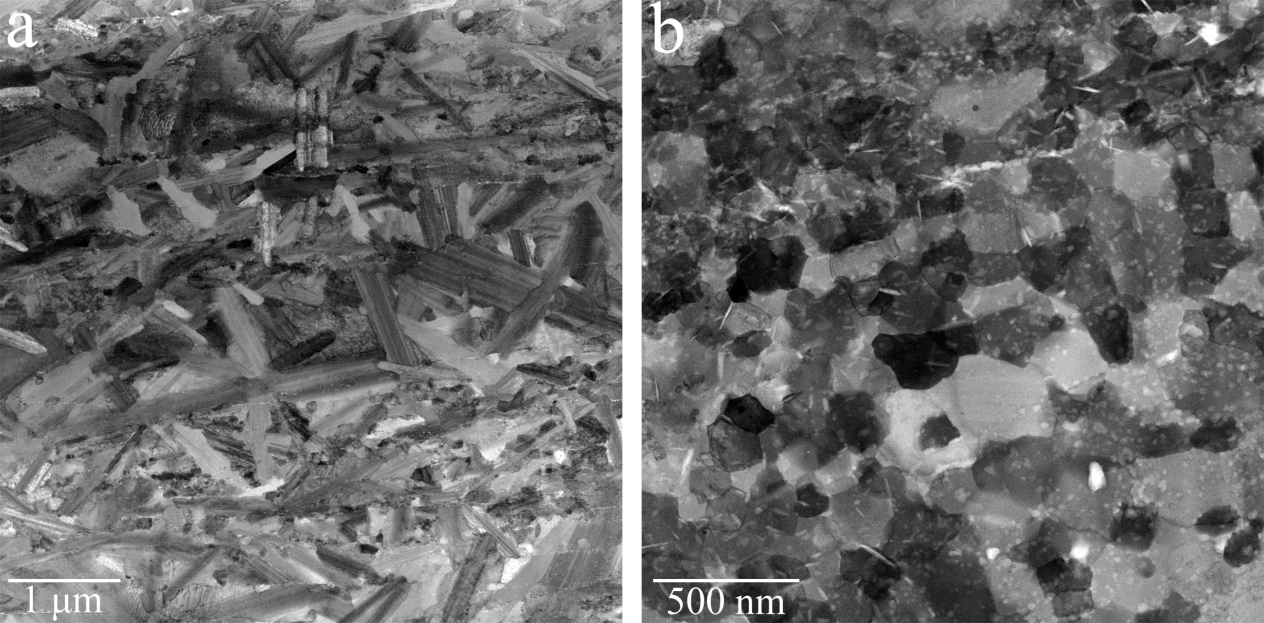


**Figure S1. BF-TEM images showing the grain structures of (a) Ni_3_X and (b) NiX.**


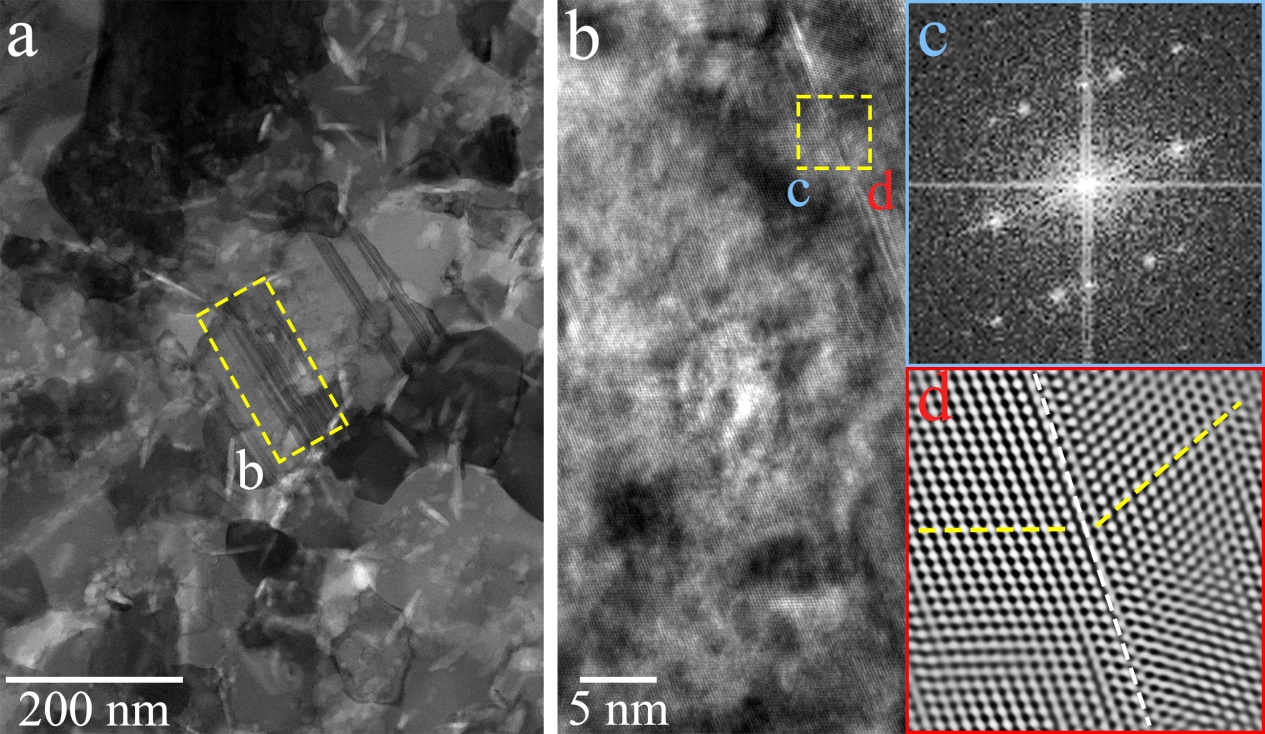


**Figure S2. Annealing nano-twins in the primary A1 grain of NiX.** (a) BF-TEM image of the grain structures. (b) HRTEM image of the rectangular region in (b). (c) FFT and (d) IFFT of the boxed region marked in (b) showing the presence of nano-twins.


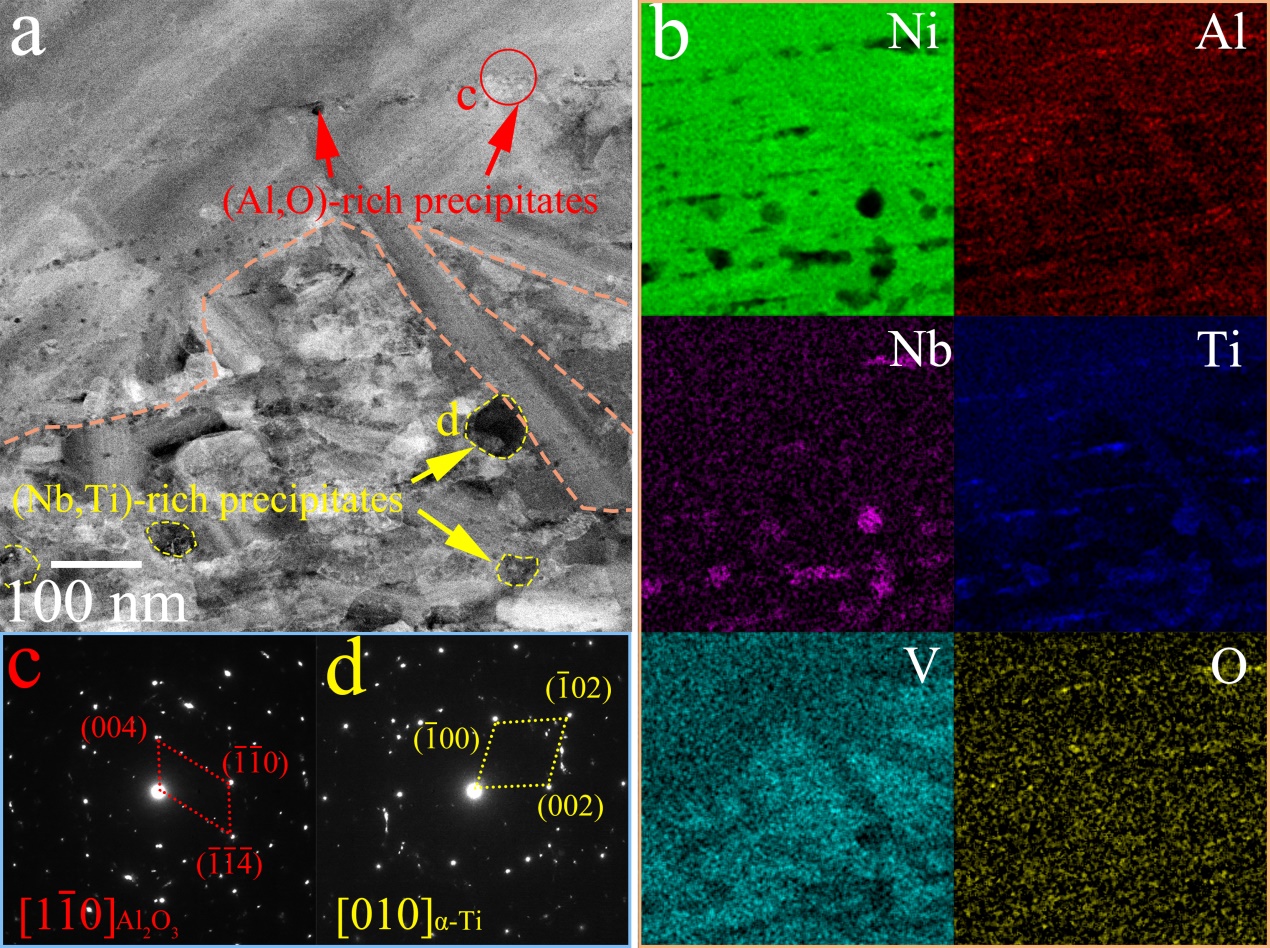


**Figure S3. Microstructure and chemical composition of two precipitates in Ni_3_X.** (a) STEM image showing the presence of two types of precipitated phases in the Ni_3_X, with grain sizes ranging from a few tens to over a hundred nanometres. The total volume fraction of both precipitates in the matrix is less than 3.5 vol.%. (b) The corresponding EDS mappings. The chemical compositions of the (Al, O)-rich and (Nb, Ti)-rich precipitates are Al_43.9_O_43.4_Ni_7.8_Ti_3.4_Nb_0.8_V_0.7_ (at.%) and Nb_44.1_Ti_35.2_Al_10.1_V_4.8_Ni_3.2_O_2.6_ (at.%), respectively. (c, d) SAED patterns indicating that (Al, O)-rich and (Nb, Ti)-rich precipitates have Al_2_O_3_-type and α-Ti-type close-packed hexagonal structure, respectively.


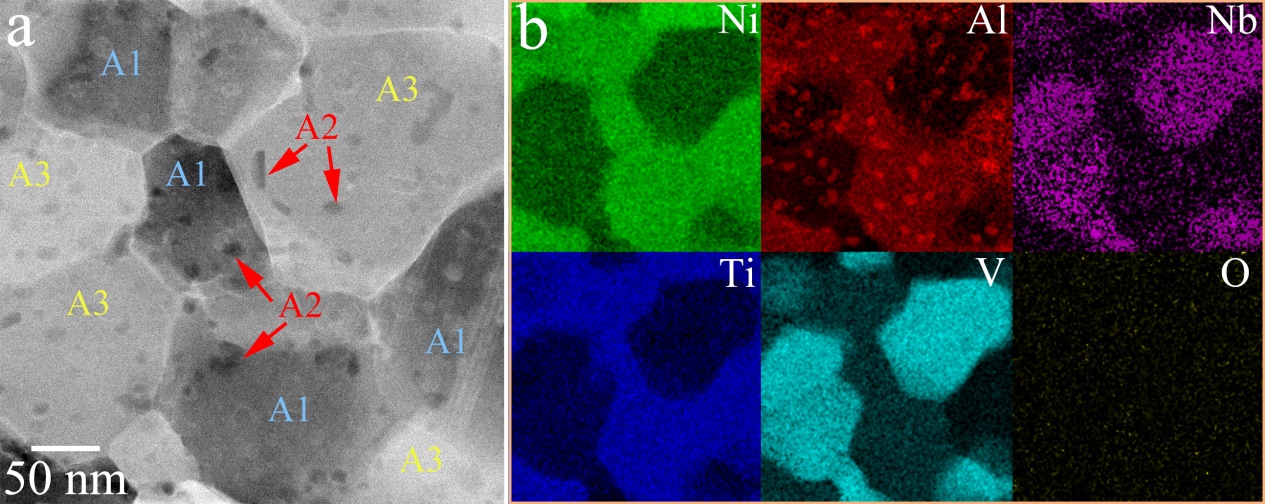


**Figure S4. Chemical composition of the nanohierarchical architecture consisting of A1-A3 nano-coupled grains and A2 nanoprecipitates in NiX.** (a) STEM image and (b) corresponding EDS mappings illustrating no additional precipitates/phases are formed in NiX.

**
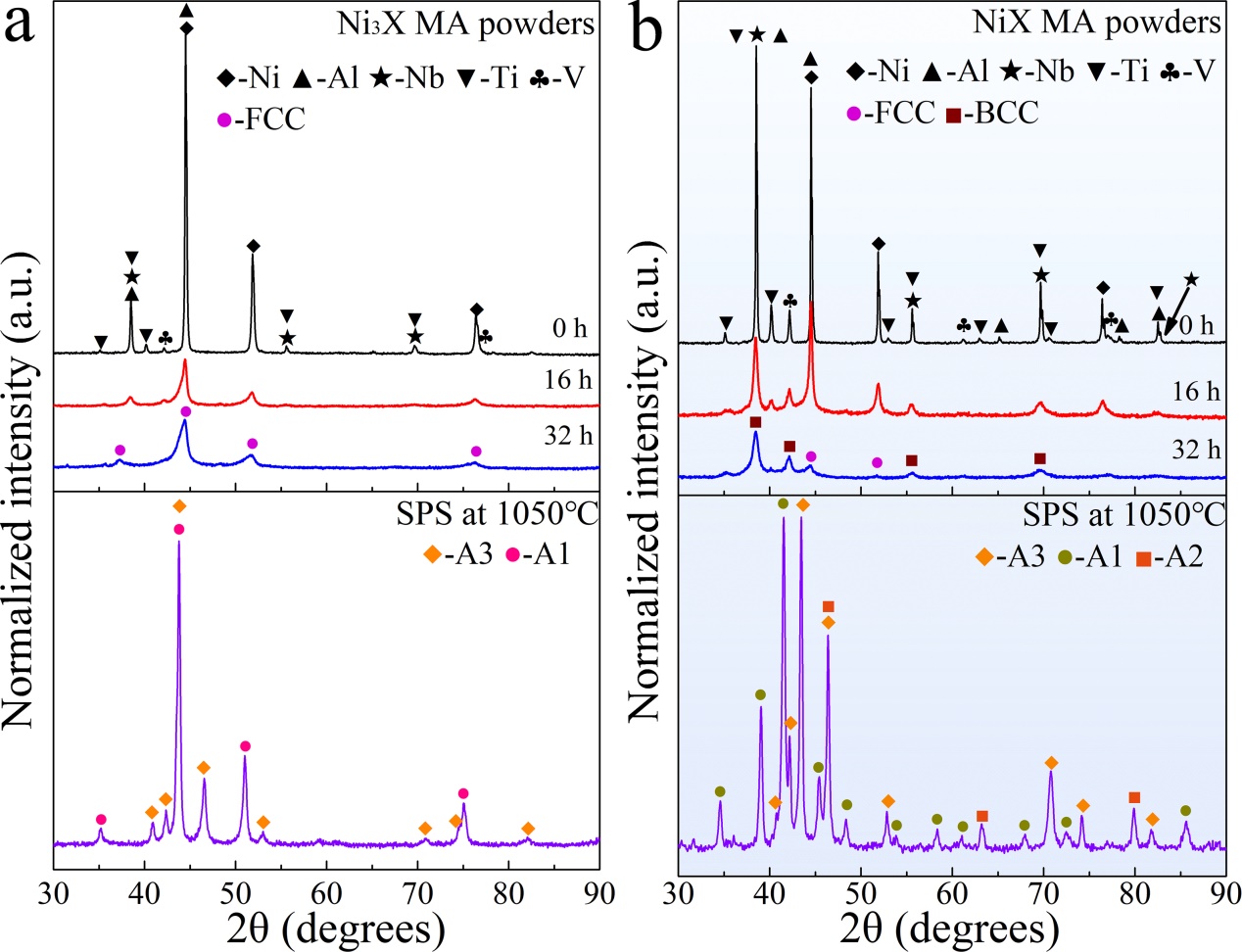
**

**Figure S5. XRD patterns of the MA powders at different milling durations and the sintered samples.** (a) Ni_3_X. (b) NiX.

**
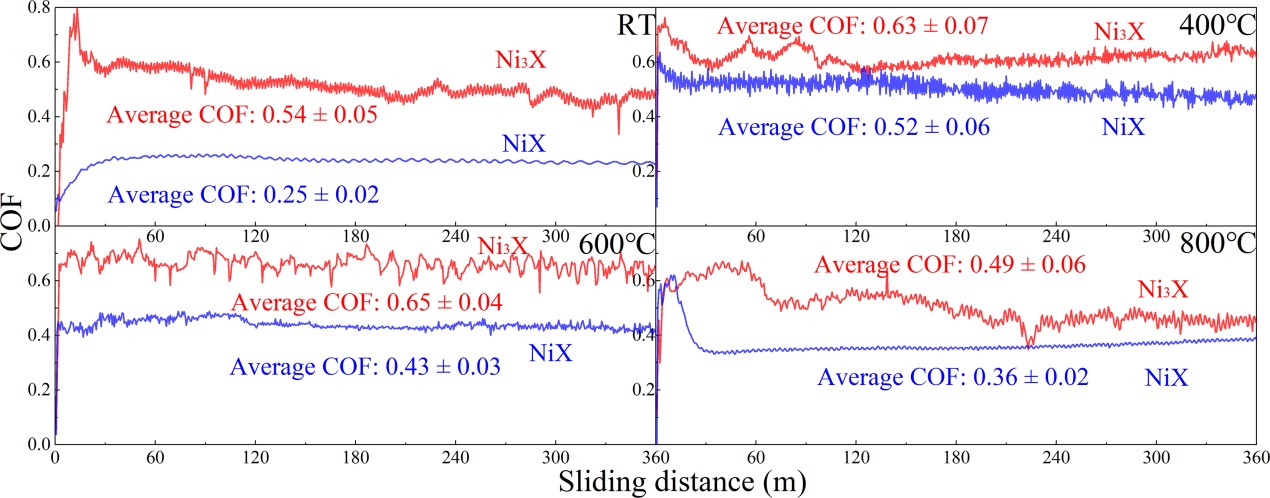
**

**Figure S6. Coefficient of friction (COF) curves of NiX and Ni_3_X obtained during sliding from RT to 800℃.**


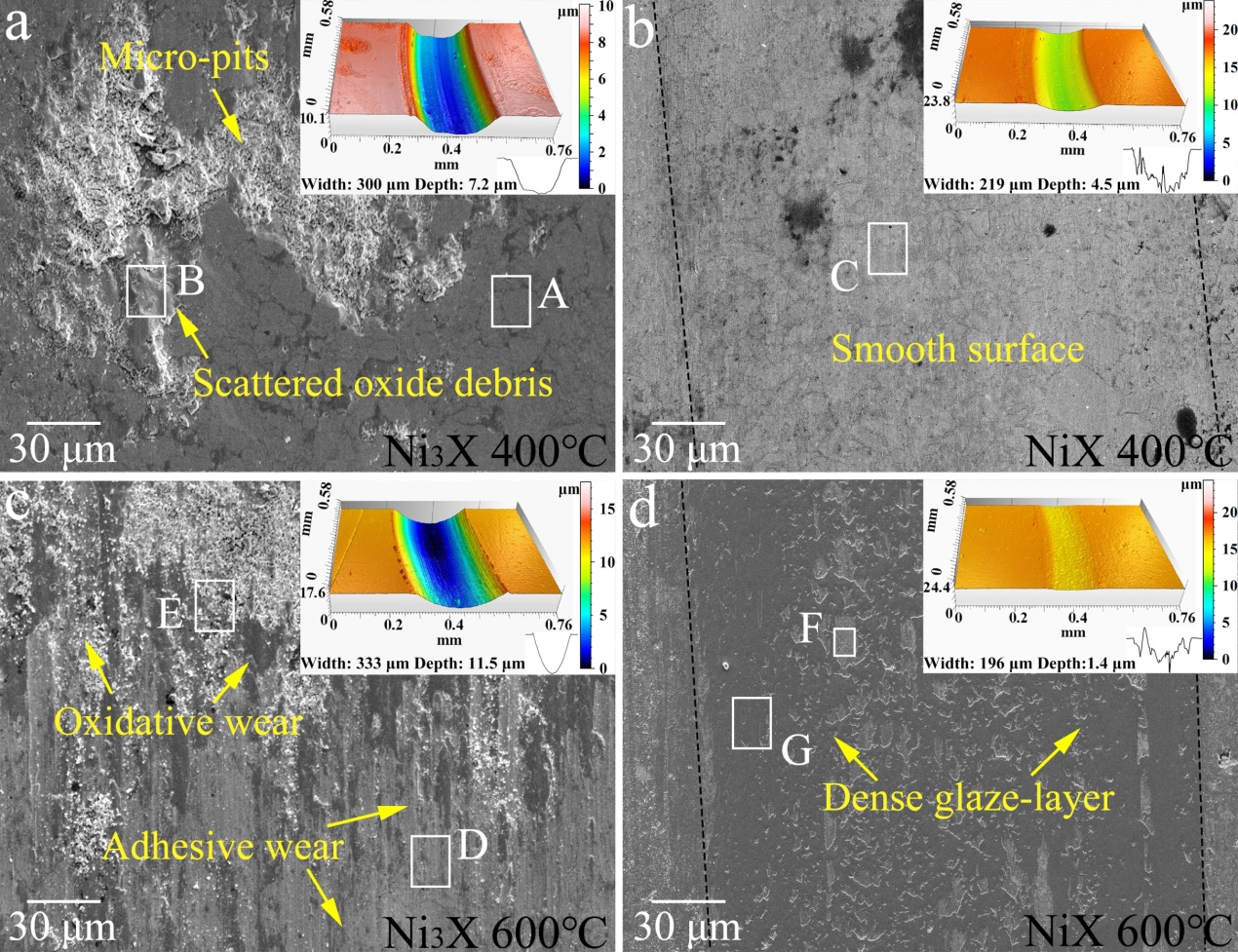


**Figure S7. Worn surface morphology of the tested CCAs at 400℃ and 600℃.** Chemical compositions of the marked regions are shown in Table S2. Inset: 3D morphologies of worn tracks.


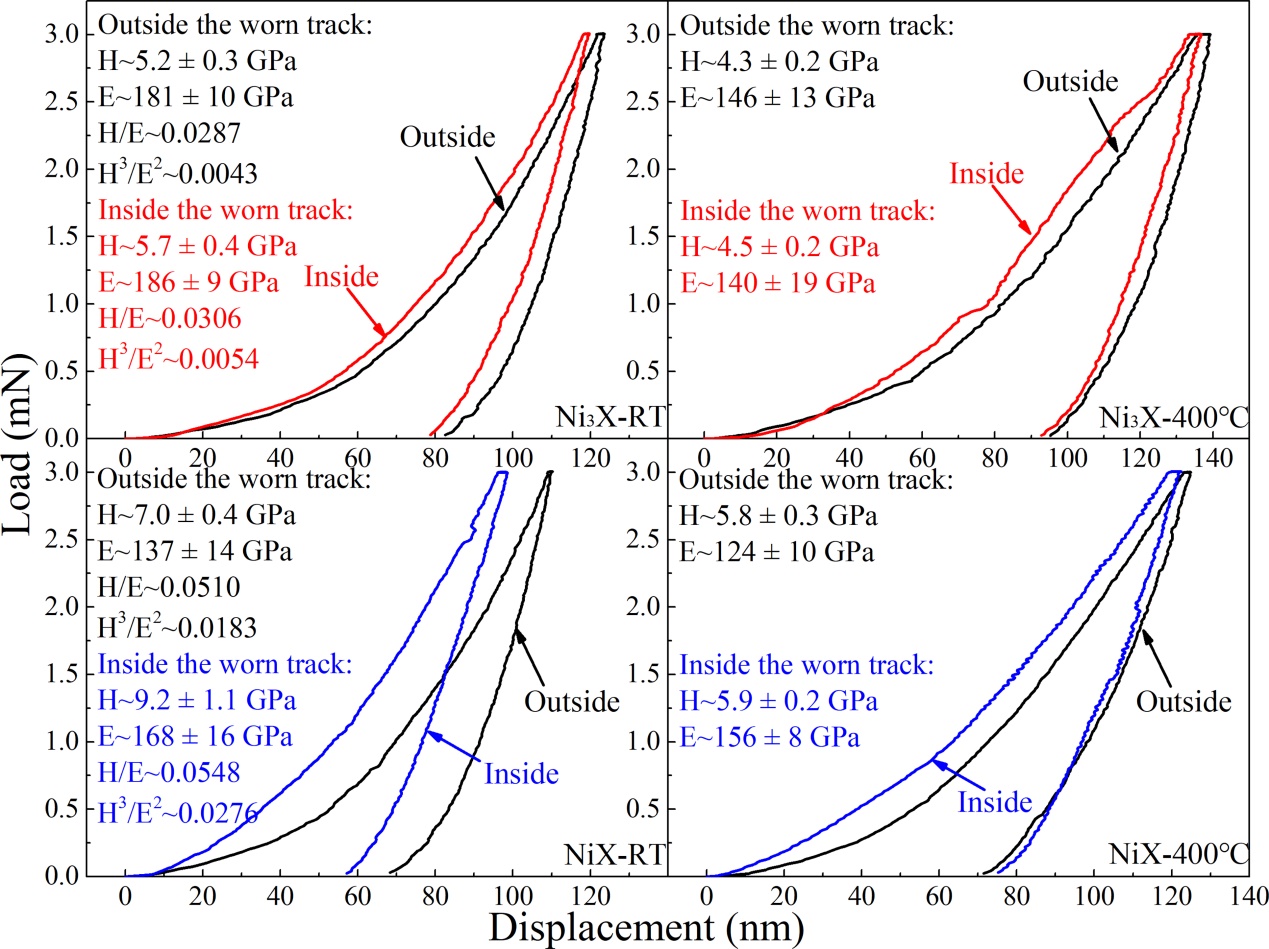


**Figure S8. Load-displacement curves of the inside and outside of the worn tracks for the tested CCAs after wear testing at RT and 400℃.** The recorded values of microhardness (H) and elastic modulus (E) are the average values obtained from at least five tests.


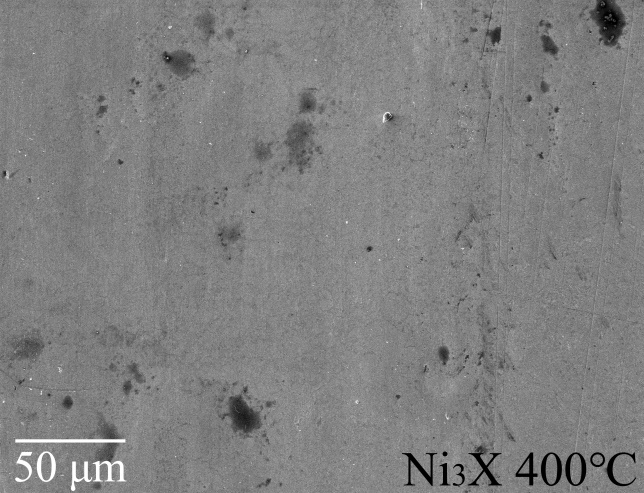


**Figure S9. SEM image of the Ni_3_X surface away from the worn track after wear testing at 400℃.**


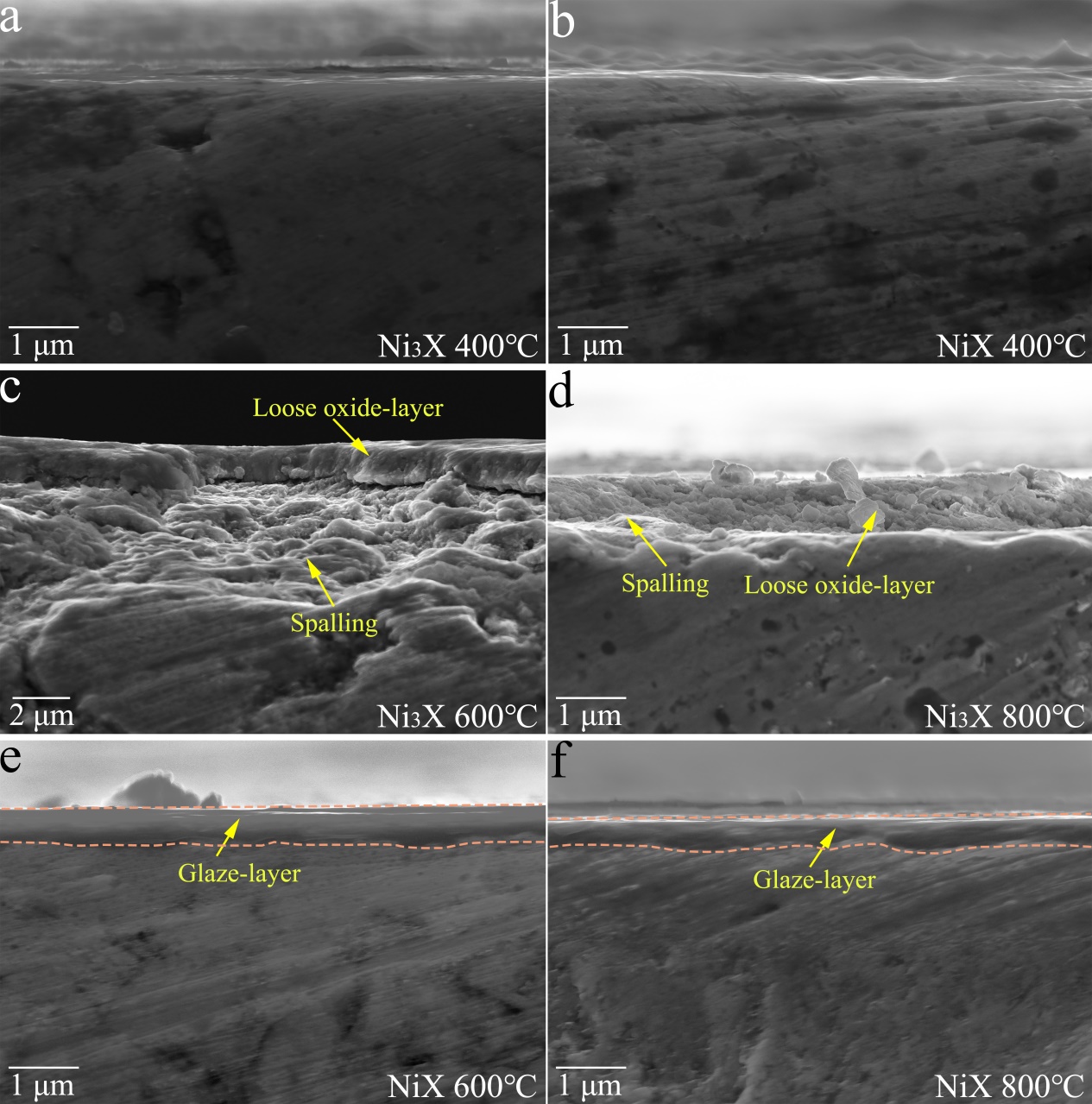


**Figure S10. Cross-sectional morphologies of worn tracks for the Ni_3_X and NiX after wear testing between 400℃ and 800℃.**


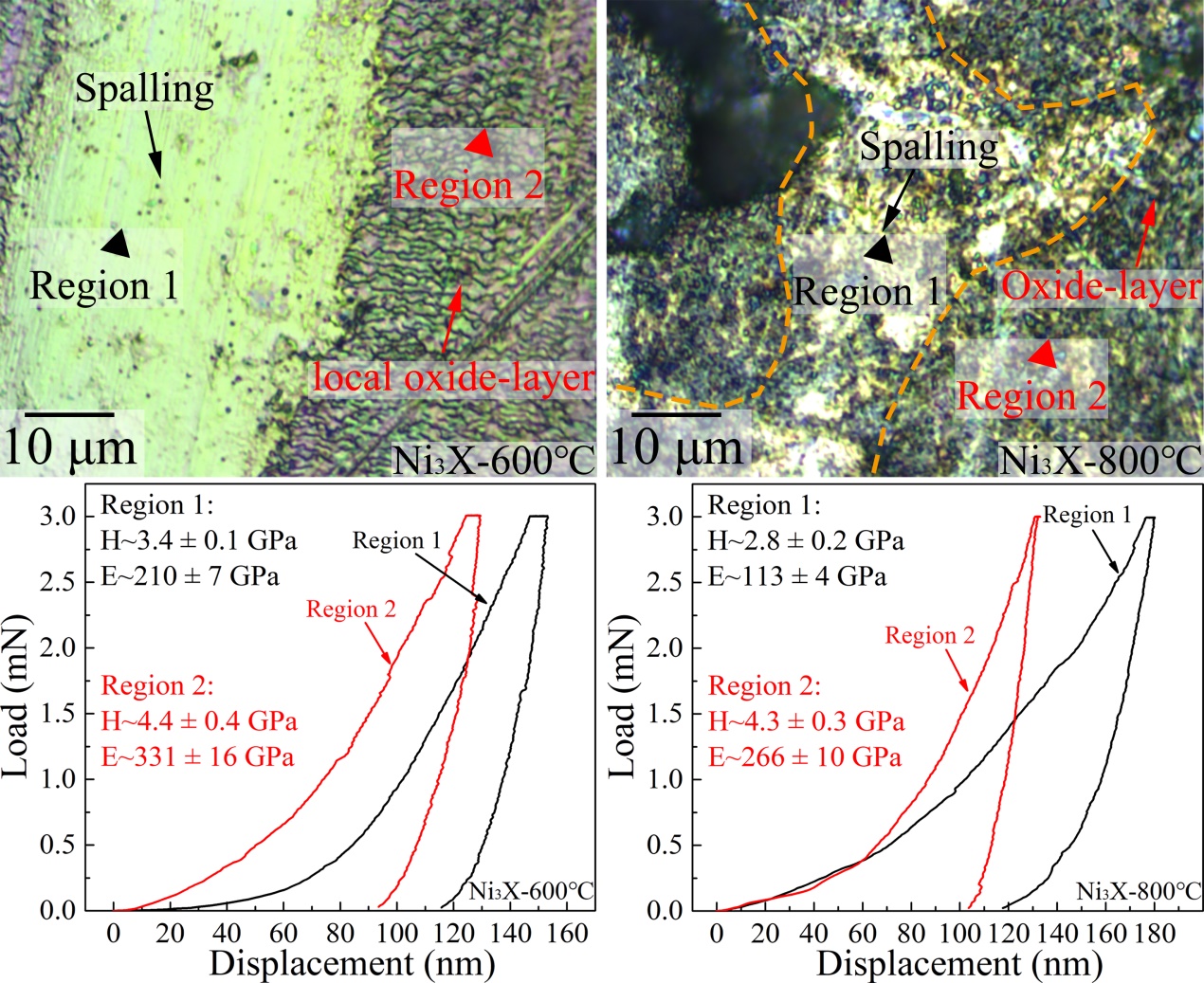


**Figure S11. Optical images of the worn surface for the tested Ni_3_X after wear at elevated temperatures, and the load-displacement curves of the corresponding regions marked therein.** The recorded values of microhardness (H) and elastic modulus (E) are the average values obtained from at least five tests.


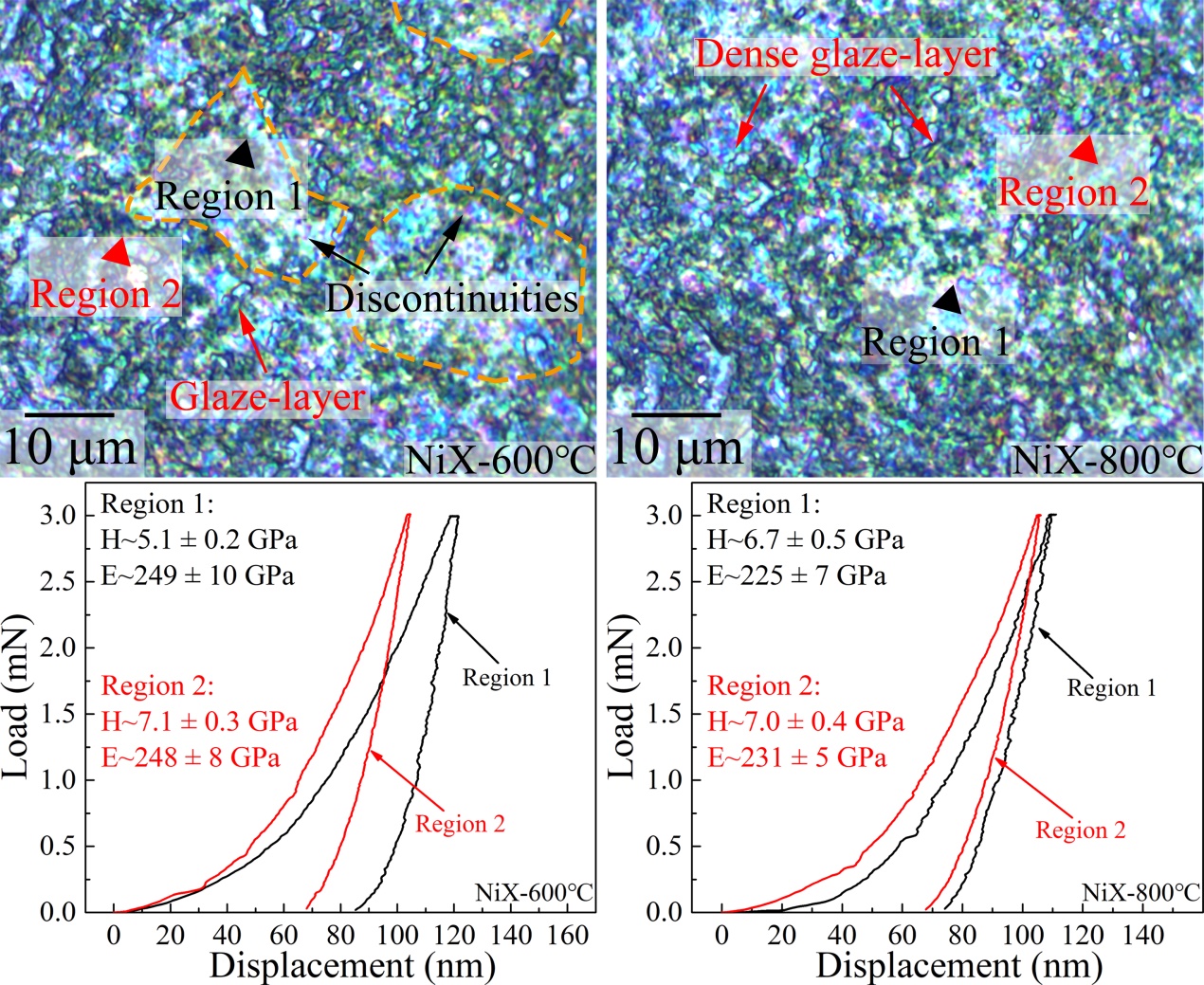


**Figure S12. Optical images of the worn surface for the tested NiX after wear at elevated temperatures, and the load-displacement curves of the corresponding regions marked therein.** The recorded values of microhardness (H) and elastic modulus (E) are the average values obtained from at least five tests.


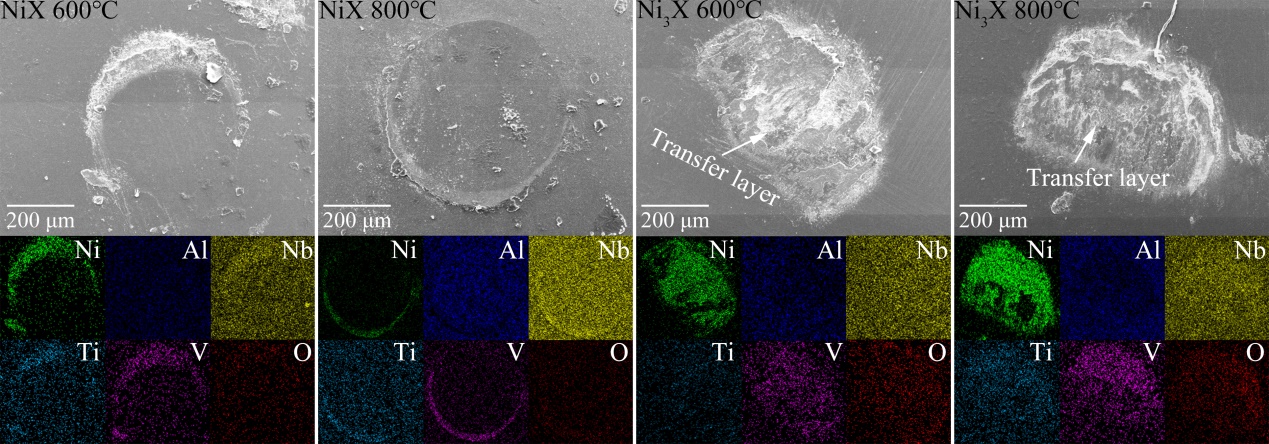


**Figure S13. Surface morphology and element distribution the Si_3_N_4_ counterbody sliding against Ni_3_X and NiX tested between 600℃ and 800℃.** The EDS mapping of the transfer layer adhered on the counterface of Si_3_N_4_ balls indicating the occurrence of the adhesive wear.

**
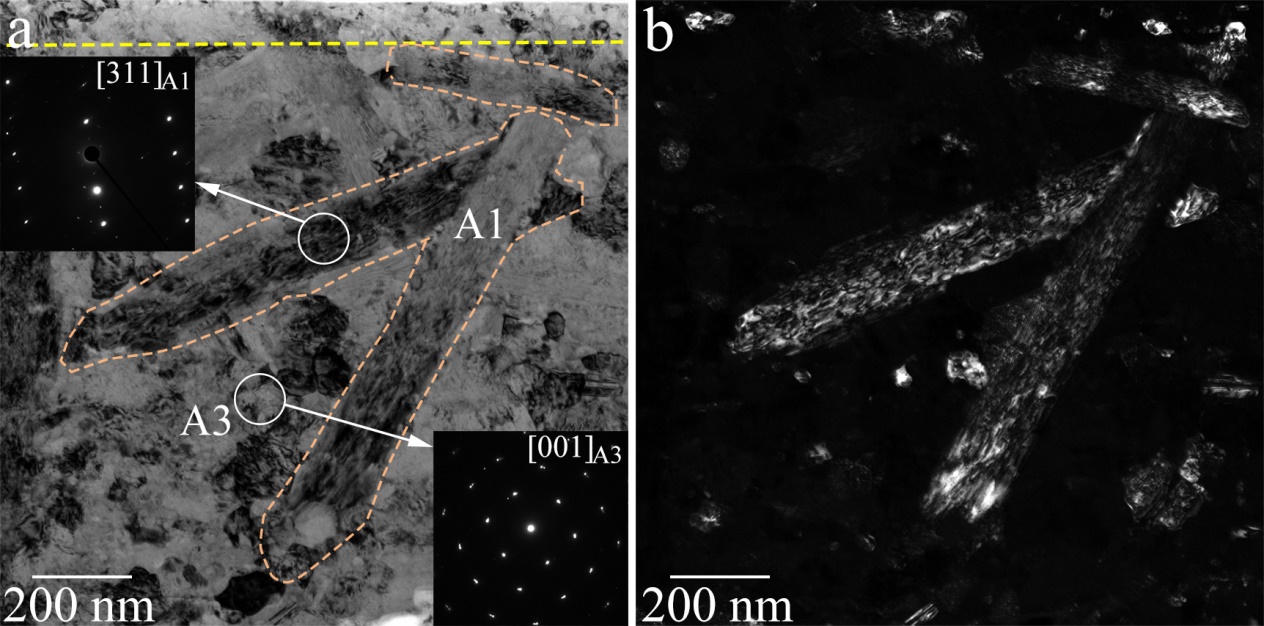
**

**Figure S14. Subsurface microstructure of Ni_3_X after wear testing at RT.** (a) BF-TEM image and corresponding SAED pattern indicating a higher density of dislocations in the lamellar A1 phase than in the equiaxed A3 phase. (b) DF-TEM image corresponding to Fig. S14a.

**
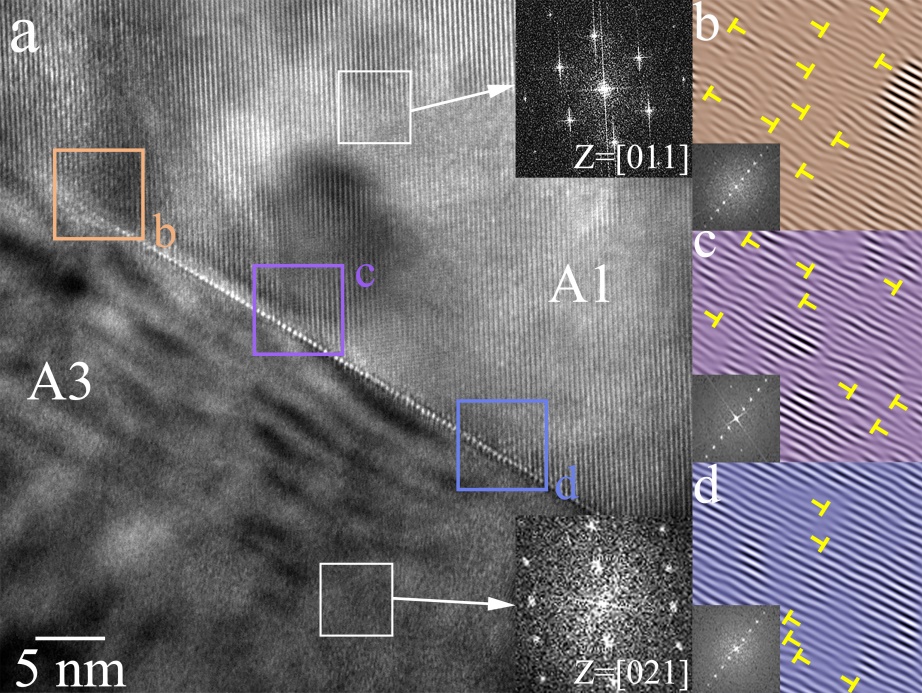
**

**Figure S15. Dislocation interactions at the interface between heterophases in the tribo-layer of NiX.** (a) HRTEM image and its corresponding (b-d) FFT/IFFT revealing the dislocation interaction near the GB of nano-coupled grains in the NC tribo-layer.

**
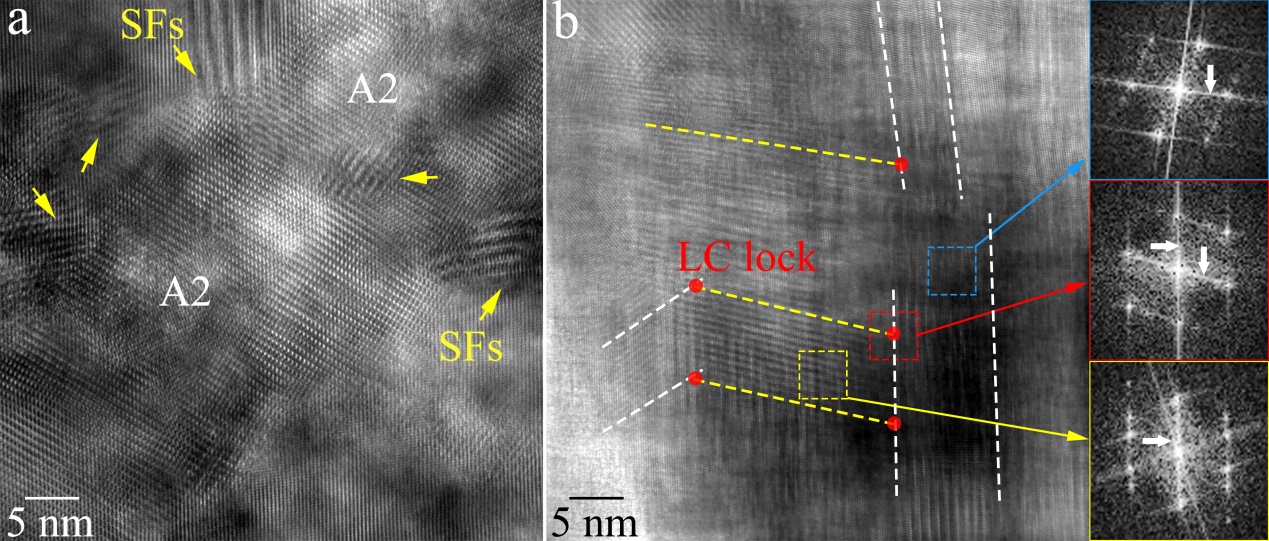
**

**Figure S16. SF network (dash line) and Lomer-Cottrell (LC) locks (red circles) around A2 phase in the UFG tribo-layer of NiX.** (a) HRTEM image of a high density of SF bundles around A2 phase. (b) HRTEM image and corresponding FFT patterns showing the profuse formation of LC locks.

**
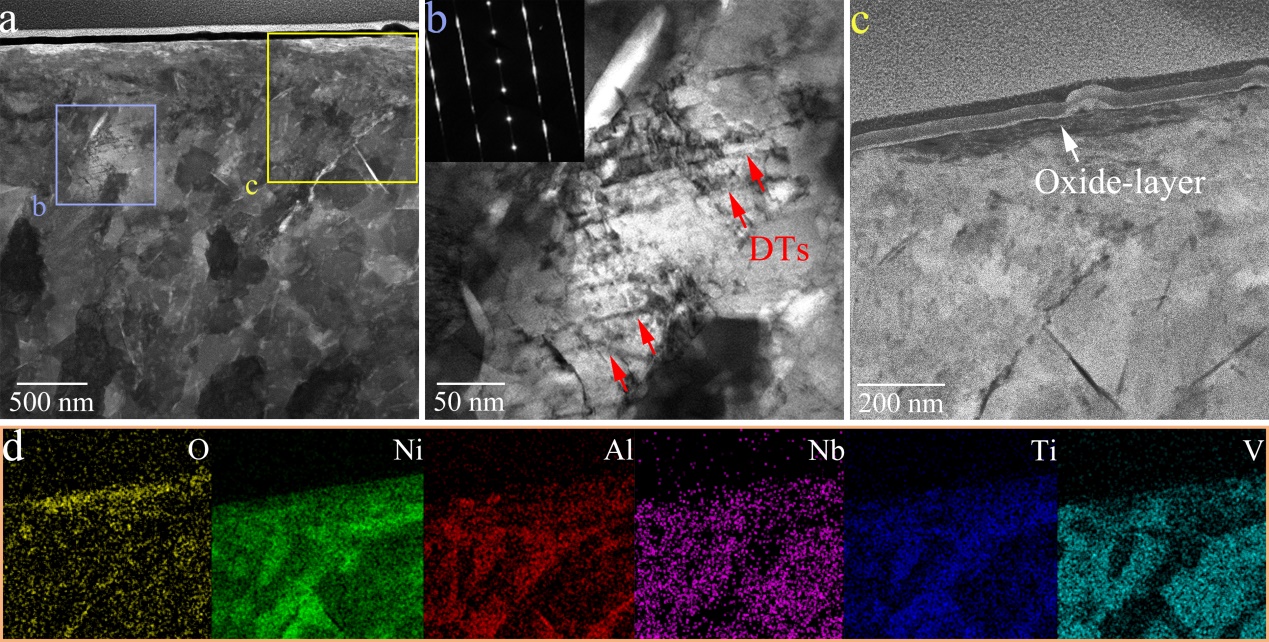
**

**Figure S17. Top worn subsurface microstructure of NiX after wear testing at RT.** (a) STEM image of worn subsurface layer. (b) High-magnification BF-TEM image of the selected deformation twins (DTs, red arrows) area and corresponding SAED pattern. (c) HAADF-STEM image of the yellow dotted box marked in Fig. S17a. The white arrow points to the nano-scale oxide-layer. (d) EDS mapping corresponding to the Fig. S17c.


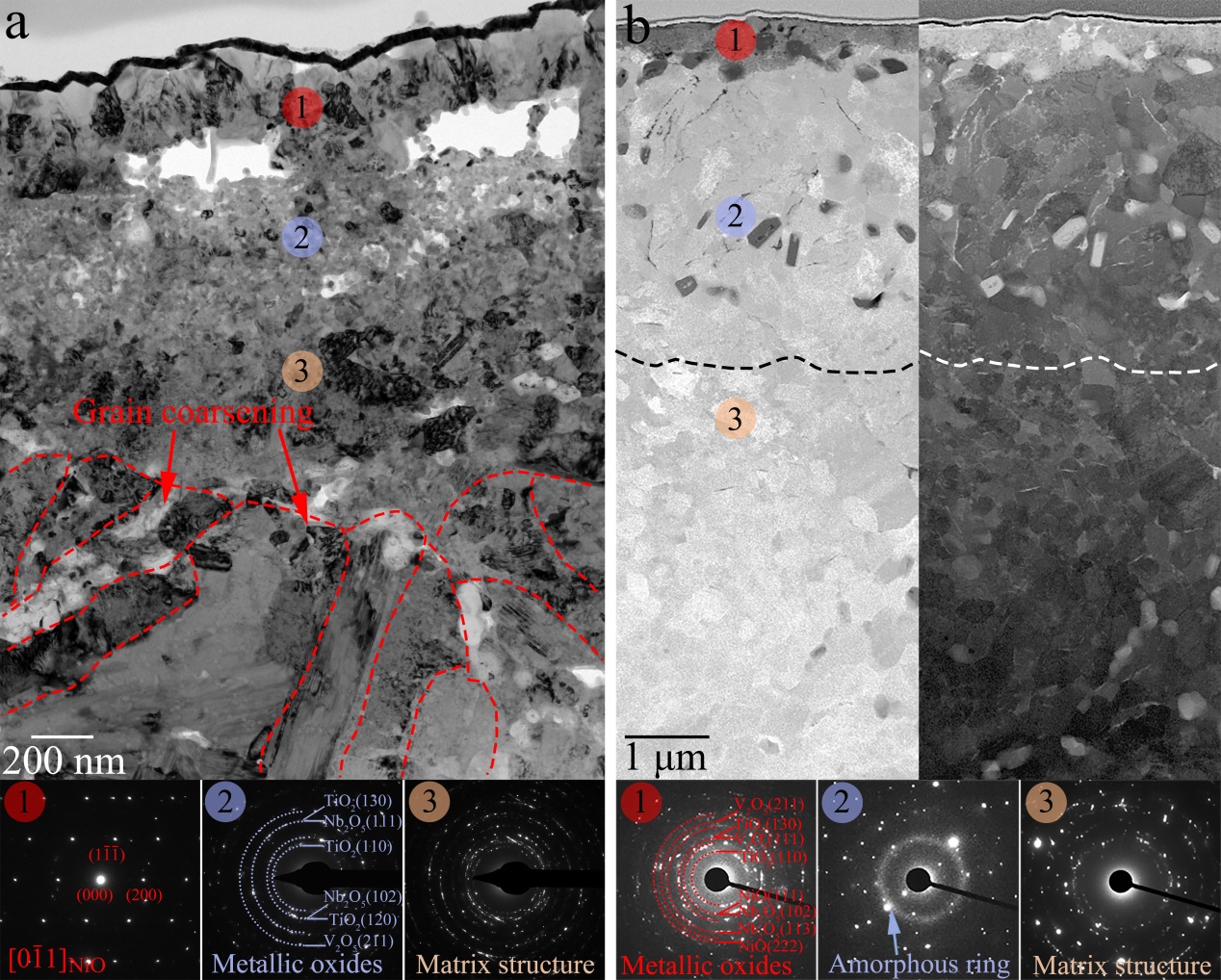


**Figure S18. Subsurface layer microstructure of Ni_3_X and NiX after wear testing at 800℃.** (a) BF-TEM image of subsurface microstructure of Ni_3_X. (b) HAADF-STEM image and corresponding BF-TEM image of subsurface microstructure of NiX. Underside: SAED patterns of the different regions marked in the subsurface layer.


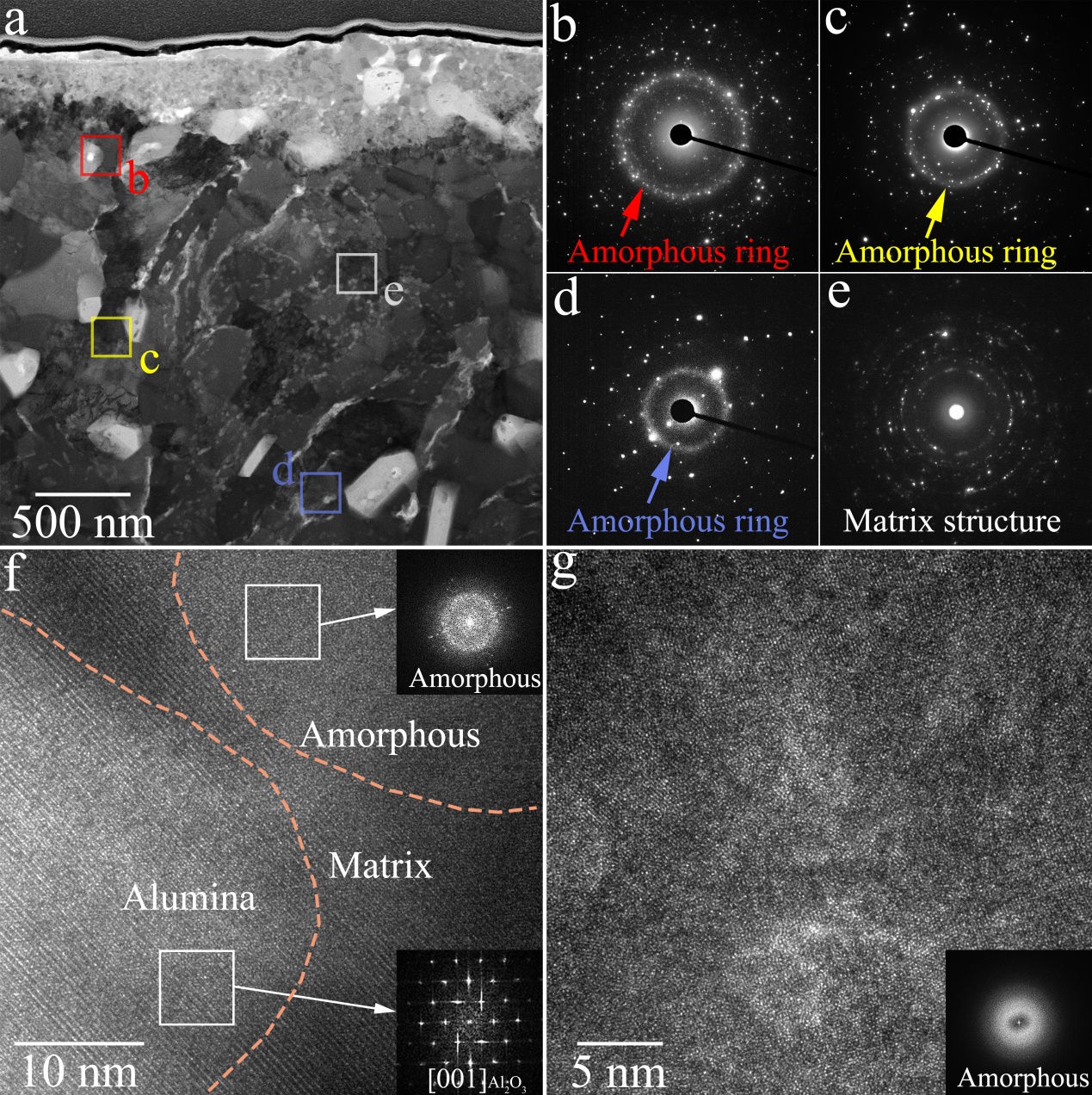


**Figure S19. Nanometer-sized amorphous oxides formed near alumina nanoparticles in the subsurface layer of NiX after wear testing at 800℃.** (a) BF-TEM image and (b-e) the corresponding SAED patterns presenting the amorphous-crystalline nanocomposites in the worn subsurface layer. (f, g) HRTEM images of the (f) red and (g) blue boxes marked in Fig. S19a or Figure 5(c).

**
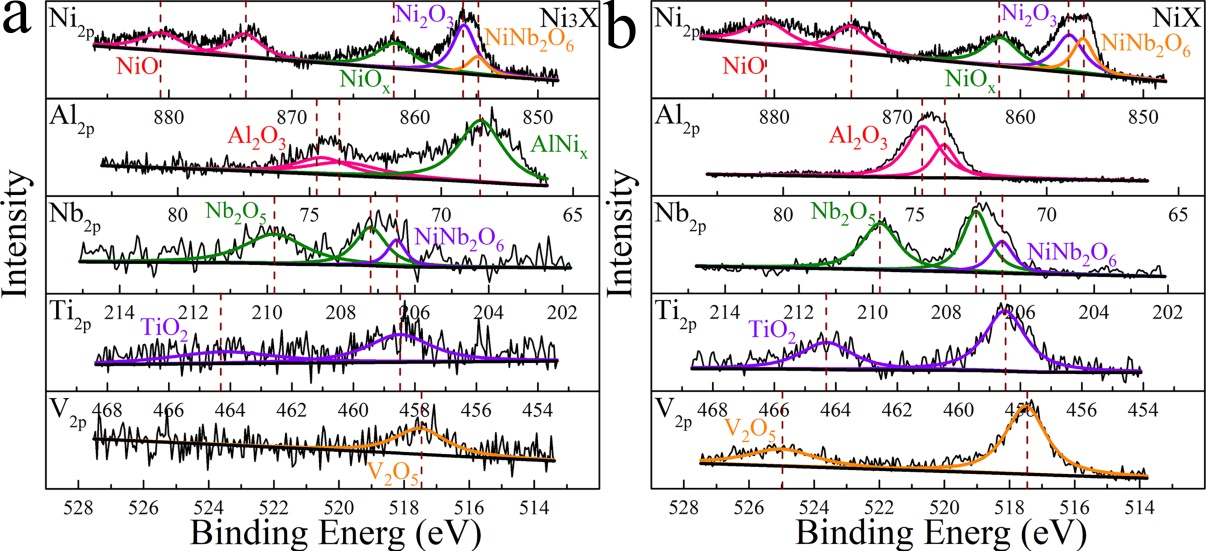
**

**Figure S20. Chemical states of the worn surfaces for CCAs after wear testing at 800℃.** (a) Ni_3_X and (b) NiX.


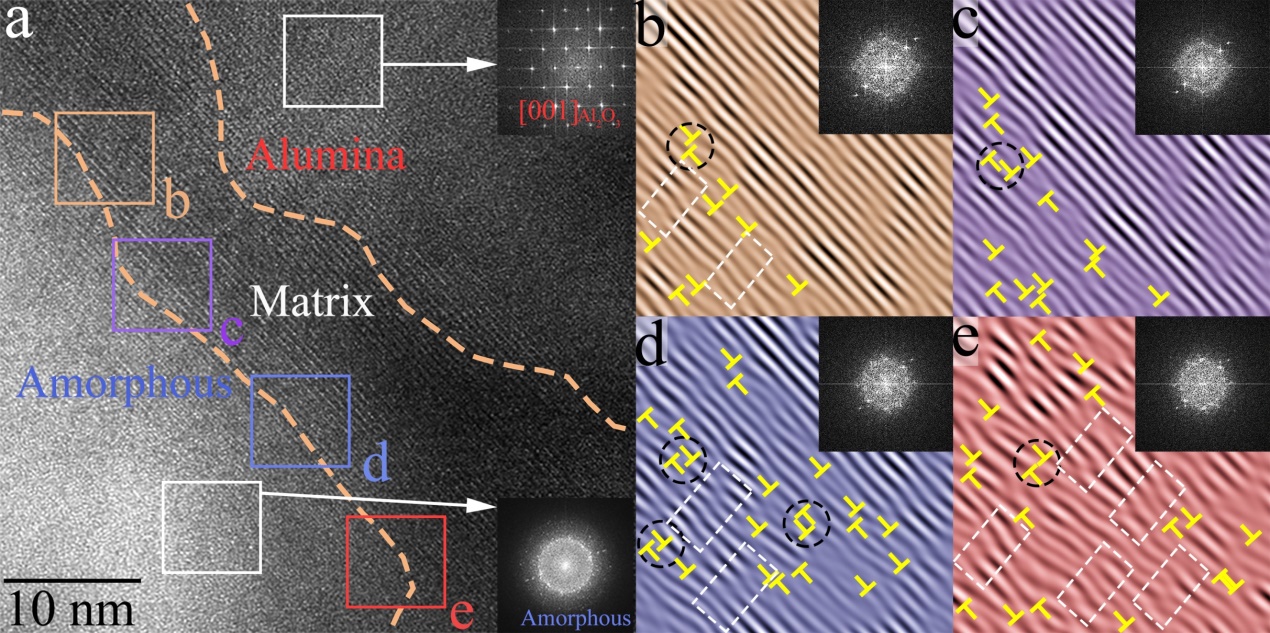


**Figure S21.** **HRTEM image of the yellow box marked in Fig. S19a or Figure 5(c).** (a) HRTEM image and (b-e) its corresponding FFT/IFFT revealing the presence of a high density of multiple dislocation activity in the interfacial region of the amorphous oxide. Insets in Fig. S21a are the corresponding FFT patterns of the amorphous phase and alumina nanoparticle. The IFFT images showing the interaction and accumulation of dislocations (in black circles) and SFs (in white boxes) at the interface.

**
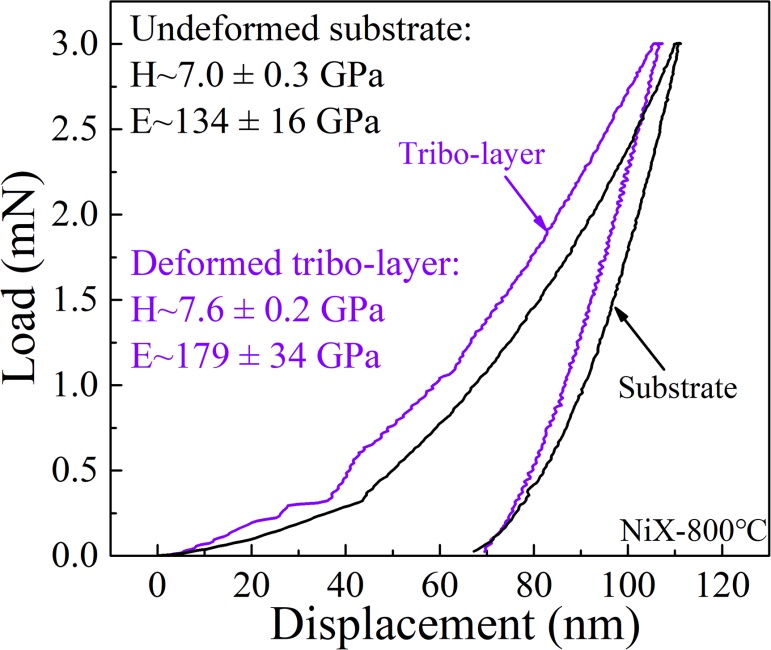
**

**Figure S22. Load-displacement curves obtained from the cross-section of the worn track of NiX after wear testing at 800℃.** Notice that the indentation locations of the deformed tribo-layer and the undeformed substrate are set at regions approximately 2 µm and 10 µm beneath the worn surface, respectively. The recorded values of microhardness (H) and elastic modulus (E) are the average values obtained from at least five tests.

**
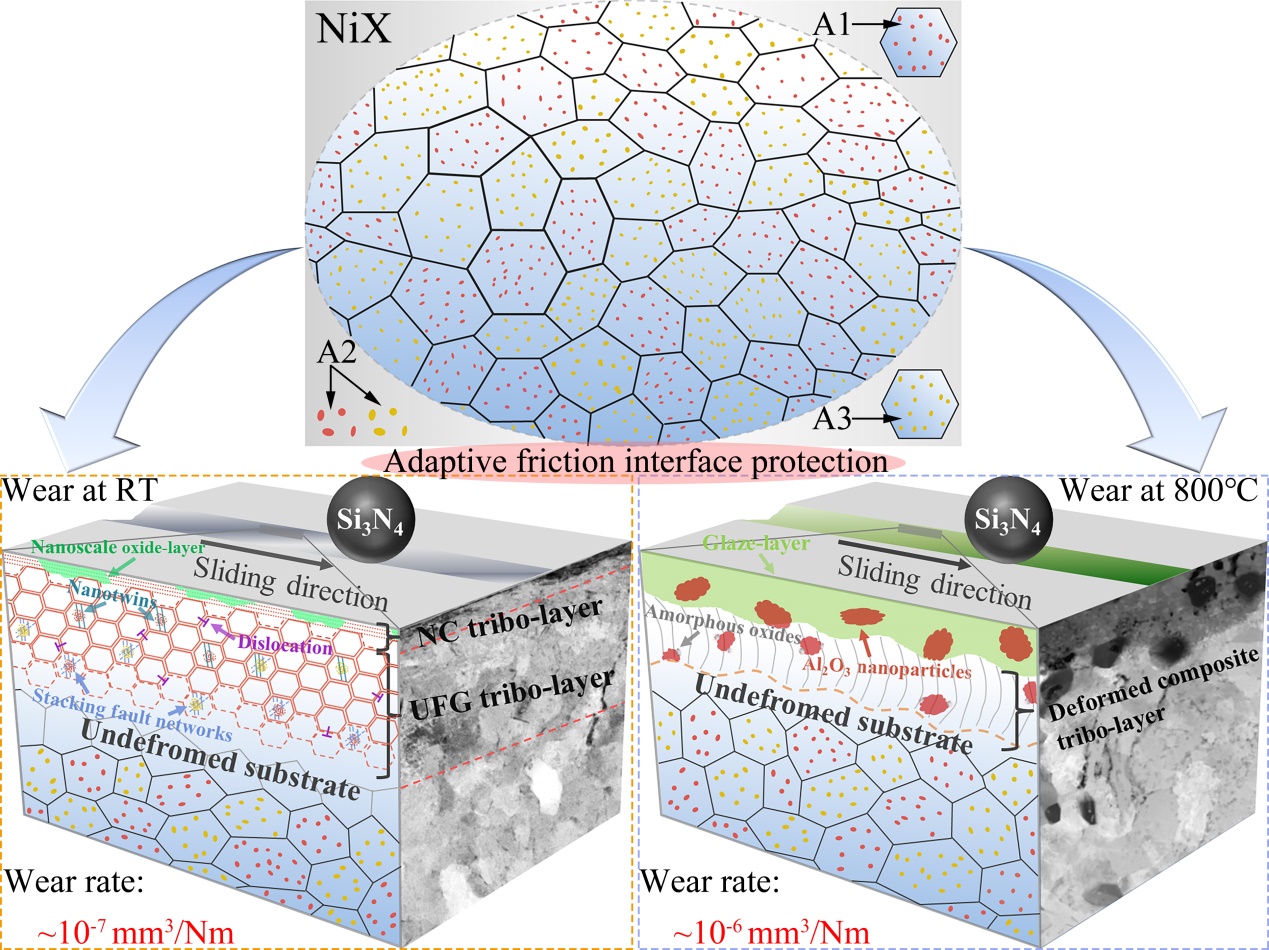
**

**Figure S23. Schematic diagram illustrating the wear mechanisms associated with adaptive friction interface protection in the NiX during sliding at RT and 800℃, respectively.**

**Table S1. STEM-EDS measured compositions of each phase in Ni_3_X and NiX.** The estimated maximum error is 1.5 at.%. The standard deviation (error) of EDS results is obtained based on at least five measured series.

| CCA | Regions/Phases +  Volume fraction (%) | Chemical composition (at.%) | | | | | |
| --- | --- | --- | --- | --- | --- | --- | --- |
|  |  | Ni | Ti | Al | Nb | V | others* |
| NiX | Nominal | 50.0 | 12.5 | 12.5 | 12.5 | 12.5 | - |
|  | Overall | 49.5 | 12.3 | 12.3 | 12.2 | 12.3 | 1.4 |
|  | A1 phase 40 vol.% | 57.7 | 18.2 | 9.3 | 6.7 | 7.3 | 0.8 |
|  | A3 phase 39 vol.% | 47.9 | 6.8 | 3.7 | 20.5 | 19.8 | 1.3 |
|  | A2 phase 21 vol.% | 38.3 | 11.6 | 31.5 | 7.6 | 8.3 | 2.7 |
| Ni_3_X | Nominal | 75.0 | 6.25 | 6.25 | 6.25 | 6.25 | - |
|  | Overall | 74.6 | 6.2 | 6.2 | 6.3 | 6.3 | 0.4 |
|  | A1 phase 74 vol.% | 76.3 | 6.2 | 6.1 | 5.8 | 5.3 | 0.3 |
|  | A3 phase 26 vol.% | 70 | 6.3 | 6.4 | 8.1 | 8.5 | 0.7 |
| * These elements were C, W and O that came from the ball-milling process. | | | | | | | |

**Table S2. Chemical composition (at.%) of the worn surfaces for NiX and Ni_3_X.** The EDS results show that the oxygen concentration on the worn surface (marked in Figures 3(b)-3(e) and Fig. S7) increases as the wear testing temperature increases, proving that tribo-oxidation is significant upon high-temperature wear.

| **Temperature** | **Region** | **Ni** | **Al** | **Nb** | **Ti** | **V** | **O** |
| --- | --- | --- | --- | --- | --- | --- | --- |
| RT | Ni_3_X-A | 69.7 | 5.8 | 5.4 | 4.9 | 5.1 | 9.1 |
|  | Ni_3_X-B | 58.1 | 8.1 | 8.3 | 7.5 | 6.6 | 11.4 |
|  | NiX-C | 44.3 | 8.7 | 7.5 | 9.8 | 11.1 | 18.6 |
| 400℃ | Ni_3_X-A | 32.3 | 11.1 | 9.6 | 10.3 | 13.8 | 22.9 |
|  | Ni_3_X-B | 28.4 | 5.2 | 6.3 | 9.6 | 9.1 | 41.4 |
|  | NiX-C | 40.7 | 6.4 | 4.2 | 7.7 | 9.4 | 31.6 |
| 600℃ | Ni_3_X-D | 32.1 | 8.4 | 10.7 | 8.9 | 12.5 | 27.4 |
|  | Ni_3_X-E | 20.1 | 7.7 | 14.1 | 8.2 | 10.7 | 39.2 |
|  | NiX-F | 33.1 | 13.3 | 14.9 | 13.5 | 13.6 | 11.6 |
|  | NiX-G | 17.3 | 11.4 | 12.8 | 7.9 | 9.7 | 40.9 |
| 800℃ | Ni_3_X-D | 35.4 | 6.7 | 9.9 | 9.1 | 7.2 | 31.7 |
|  | Ni_3_X-E | 31.6 | 4.6 | 7.7 | 5.2 | 5.3 | 45.6 |
|  | NiX-F | 22.7 | 8.9 | 7.8 | 8.7 | 12.4 | 39.5 |
|  | NiX-G | 17.1 | 7.8 | 10.2 | 9.6 | 6.2 | 49.1 |

**Table S3. Comparison of wear-resistance for the current CCAs with other reported high-performance bulk alloys and composites.** It should be noted that the reference materials are compared with our CCAs under similar wear test conditions. The wear rates for all materials are calculated by dividing the wear volume by the applied load and sliding distance.

| **Materials** | **Tested conditions** | **Wear rate**  **(10^-6^ mm^3^/Nm)** |
| --- | --- | --- |
| NiX  this work | RT~800℃; Load 5 N; Counterpart Si_3_N_4_ ball;  Sliding speed 0.2 m/s; Sliding distance 360 m. | 0.57~9.8 |
| Ni_3_X  this work | RT~800℃; Load 5 N; Counterpart Si_3_N_4_ ball;  Sliding speed 0.2 m/s; Sliding distance 360 m. | 11.0~42.0 |
| Q235 low carbon steel Ref. [40] | RT~500℃; Load 5 N; Counterpart ZrO_2_ ball;  Sliding speed 0.16 m/s; Sliding distance 94 m. | 17.4~83.1 |
| 316L stainless steel  Ref. [41] | RT~600℃; Load 5 N; Counterpart Al_2_O_3_ ball;  Sliding speed 0.03 m/s; Sliding distance 113 m. | 320.0~660 |
| AISI 52100 bearing steel Ref. [42] | RT~500℃; Load 5 N; Counterpart Al_2_O_3_ ball;  Sliding speed 0.3 m/s; Sliding distance 1080 m. | 11.4~14.5 |
| AISI 8620 bearing steel Ref. [42] | RT~500℃; Load 5 N; Counterpart Al_2_O_3_ ball;  Sliding speed 0.3 m/s; Sliding distance 1080 m. | 14.1~18.1 |
| Ni_3_Al Ref. [43] | RT~800℃; Load 10 N; Counterpart Si_3_N_4_ ball;  Sliding speed 0.2 m/s; Sliding distance 1123 m. | 200~490 |
| NiAl Ref. [20] | RT~800℃; Load 20 N; Counterpart Al_2_O_3_ ball;  Sliding speed 0.11 m/s; Sliding distance 378 m. | 20.0~200 |
| Ni_3_Al-Ag Ref. [46] | RT~800℃; Load 10 N; Counterpart Si_3_N_4_ ball;  Sliding speed 0.2 m/s; Sliding distance 360 m. | 70.0~210 |
| Ni_3_Al-CrMo-ZrB-Ag Ref. [46] | RT~800℃; Load 10 N; Counterpart Si_3_N_4_ ball;  Sliding speed 0.2 m/s; Sliding distance 360 m. | 41.0~200 |
| Ni_3_Al-Cr-CaF_2_/  BaF_2_-Ag Ref. [47] | RT~800℃; Load 20 N; Counterpart Si_3_N_4_ ball;  Sliding speed 0.19 m/s; Sliding distance 228 m. | 20.0~250 |
| NiAl-CrMo-CaF_2_  -Ag Ref. [48] | RT~800℃; Load 10 N; Counterpart Si_3_N_4_ ball;  Sliding speed 0.2 m/s; Sliding distance 360 m. | 65.0~410 |
| NiAl-CrMo-ZnO  Ref. [44] | RT~800℃; Load 10 N; Counterpart Si_3_N_4_ ball;  Sliding speed 0.19 m/s; Sliding distance 684 m. | 44~230 |
| NiAl-PbO  Ref. [45] | RT~800℃; Load 10 N; Counterpart Si_3_N_4_ ball;  Sliding speed 0.2 m/s; Sliding distance 960 m. | 40.0~105 |
| CoCrFeNi HEA  Ref. [21] | RT~800℃; Load 5 N; Self-mated pin;  Sliding speed 0.17 m/s; Sliding distance 306 m. | 60.0~550 |
| CoCrFeNiAl_0.25_ HEA Ref. [19] | RT~600℃; Load 10 N;Counterpart Si_3_N_4_ ball;  Sliding speed 0.08 m/s; Sliding distance 144 m. | 150~355 |
| CoCrFeNiAl_0.6_ HEA Ref. [3] | RT~600℃; Load 10 N;Counterpart Si_3_N_4_ ball;  Sliding speed 0.13 m/s; Sliding distance 227 m. | 45.0~515 |
| AlCoCrFeNi HEA  Ref. [17] | RT~800℃; Load 5 N;Counterpart SiC ball;  Sliding speed 0.2 m/s; Sliding distance 360 m. | 15.0~198 |
| HfTaTiVZr/TaTiVWZr HEAs Ref. [11] | RT~450℃; Load 50 N; Counterpart Si_3_N_4_ ball;  Sliding speed 0.04 m/s; Sliding distance 190 m. | 220~300/  100~280 |
| AlCoCrFeNi-hBN-Ag composite Ref. [17] | RT~800℃; Load 5 N;Counterpart SiC ball;  Sliding speed 0.2 m/s; Sliding distance 360 m. | 30.0~98.0 |
| CoCrFeNi-MoS_2_-Gr  composite Ref. [22] | RT~800℃; Load 5 N;Counterpart Si_3_N_4_ ball;  Sliding speed 0.28 m/s; Sliding distance 504 m. | 40.0~75.0 |

**Supplementary Note 1. Estimate of the Hertzian contact stress and flash contact temperature during wear test at RT.**

According to the Hertzian model [8], the elastic contact stress of the compressed tested CCA samples is distributed in the plane strain field between the friction interface of tribo-pairs. Note that subscripts 1 and 2 in the following formulas represent tested NiX sample and counterpart ball, respectively. The maximum applied stress $\sigma_{max}$ and the contact radius *α_A_* are written as

$\sigma_{max}=\frac{3P}{2\pi{\alpha_{A}}^{2}}$ (1)

$\alpha_{A}=\sqrt[3]{\frac{3PR}{4E^{*}}}$ (2)

$\frac{1}{E^{*}}=\frac{1-{\vartheta_{1}}^{2}}{E_{1}}+\frac{1-{\vartheta_{2}}^{2}}{E_{2}}$ (3)

where *P* is the normal applied load, *R* is the counterpart ball radius, E and ϑ represent the elastic modulus and Poisson ratio, respectively. The E1 of the NiX CCA is about 137 GPa that obtained from the nanoindentation tests. The E_2_ and ϑ_2_ of the commercial Si_3_N_4_ ball are 320 GPa and 0.26, respectively, which are provided by the manufacturer and verified by the material handbook. Note that the contact radius calculated from the equation (Eq) 2 is ~56 μm, which actually underestimates the maximum applied stress at frictional junction. In current work, the deformation regime during wear tests ranged from the elastic-plastic to the fully plastic, so relying solely on the elastic model to estimate the contact radius is inaccurate. We define the value of the contact radius from the cross-section of the 3D profile of worn track. The contant radius of the NiX at the frictional junction is measured to be stable at about 33 μm after sliding for 1200, 3600 and 5400 cycles. Thus, the actual contact radius should be 33 μm. The normal stresses along the depth (*σ_z_, z* axis), circumferential (*σ_θ_*) and radial (*σ_R_*) directions, respectively, are given by

$\sigma_{z}=-\sigma_{max}\times\left( 1+\frac{z^{2}}{{\alpha_{A}}^{2}} \right)^{-1}$ (4)

$\frac{\sigma_{\theta}}{\sigma_{max}}=\frac{\sigma_{R}}{\sigma_{max}}=\frac{1}{2}\left( 1+\frac{z^{2}}{{\alpha_{A}}^{2}} \right)^{-1}-\left( 1+\vartheta_{1} \right)\left[ 1-\frac{z}{\alpha_{A}}\tan^{-1} \left( \frac{\alpha_{A}}{z} \right) \right]$ (5)

Combining Eqs 4 to 5 and based on the von Mises yield criterion [45], the stress (*σ_x_*) at any location in the depth direction of the stress field can be calculated by

$\frac{\sigma_{x}}{\sigma_{max}}=\frac{3}{2}\left( 1+\frac{z^{2}}{{\alpha_{A}}^{2}} \right)^{-1}-\left( 1+\vartheta_{1} \right)\left[ 1-\frac{z}{\alpha_{A}}\tan^{-1} \left( \frac{\alpha_{A}}{z} \right) \right]$ (6)

The instantaneous flash contact temperature at the frictional junction (*T_c_*), which is approximately equal to the sum of the test temperature (*T_test_*) and the temperature increment (*ΔT_max_*) due to frictional heating for Hertzian contact, can be expressed as follows

$T_{C}=T_{test}+\Delta T_{max}$ (7)

$\Delta T_{max}=\frac{2.31\alpha_{A}{}_{C}\bar{\sigma}V}{\sqrt{\pi}(\lambda_{1}\sqrt{{1.2344+P}_{e1}}+\lambda_{2}\sqrt{1.2344+P_{e2}})}$ (8)

where *μ_C_*, $\overline{\sigma}$, *V* and *λ* is COF, average contact stress (*P/*$\alpha_{A}$*^2^*), sliding speed, and thermal conductivity, respectively. The Peclet number *P_e1_* (*P_e2_=0*) is described as

$P_{e1}=\frac{V_{1}\alpha_{A}\rho_{1}C_{1}}{2\lambda_{1}}$ (9)

where $\rho_{1}$ and $C_{1}$ are the density and specific heat of tested NiX, respectively. All of relevant experimental parameters mentioned above are summarized in the following Table:

| **Material property** | NiX sample | Si_3_N_4_ ball |
| --- | --- | --- |
| *ρ*, density (kg/m^3^) | 6790 | 3220 |
| *λ*, thermal conductivity (Wm^-1^K^-1^) | 10.5 | 27.5 |
| *C*, specific heat (Jkg^-1^K^-1^) | 496 | 900 |
| *ϑ*, Poisson ratio | 0.29 | 0.26 |
| *E,* elastic modulus (GPa) | 137 | 320 |
| *α_A_*, contact radius (μm) | 33 | 33 |
| *σ_max_*,  maximum applied stress (GPa) | 2.2 | - |
| *μ_C_*,  coefficient of friction | 0.25 | - |
| *T_c_*,  flash contact temperature (K) | 511 (240℃) | 511 (240℃) |
| *Z_max_,* maximum depth (μm) | 26.7 | 26.7 |

**Supplementary Note 2. Theoretical calculations of yield strength increments along the worn subsurface layer of NiX after wear at RT.**

For the dual-phase grain structured materials, dislocations initially start to propagate inside the soft phase and pile-up at interphase boundaries/GBs [29]. The yield occurs when the leading dislocation in a pile-up can overcome the barrier strength and transmit slip across the GB. Thus, in our A1-A3 nano-coupled architecture, the strength is dominated by the size of the soft A1 phase, and the contribution of the A2 hierarchical nanoprecipitates should be neglected since its size remains constant during wear at RT. If the average grain size of subsurface layer changes from *d_1_* to *d_2_* under tribological loading, according to the Hall-Petch relationship [50], a variation/increment of the yield stress would result and can be described as

$${\Delta\sigma}_{G}=K_{f}({d_{2}}^{-\frac{1}{2}}-{d_{1}}^{-\frac{1}{2}})$$

where the Hall-Petch coefficient *K_f_* can be calculated by:

$$K_{f}={(\frac{n^{2}G^{2}b^{2}}{8d})}^{\frac{1}{2}}$$

where *n* = ~2.1 is the number of dislocations crossing the GB (obtained through HRTEN images), *G* = 76.9 GPa is the shear modulus of the FCC phase [51], $b=\sqrt{2}a/2=0.9078 \mathrm{nm}$ is the magnitude of the Burgers vector of the A1 phase, *a* is the lattice parameter of the A1 phase obtained from XRD, *d* = ~176 nm is the is the average grain size of the nano-coupled grains. As such, the variations of average grain size and yield stress along the depth from the worn surface are shown in Figures 4(c) and 4(d), respectively.
